# Chaotic map based efficient anonymous authentication and key agreement scheme for VANETS

**DOI:** 10.1371/journal.pone.0357045

**Published:** 2026-09-02

**Authors:** Dulam Devee SivaPrasad, Azees Maria

**Affiliations:** Department of Networking and Security, School of Computer Science and Engineering, VIT-AP University, Beside AP Secretariat, Amaravati, Andhra Pradesh, India; Civil Aviation University of China, CHINA

## Abstract

Vehicular Ad Hoc Networks (VANETs) have been developed as an important technology for improving road safety and traffic efficiency in intelligent transport systems. However, due to the increase in the number of cyberattacks, they have become vulnerable to several security threats that must be addressed. Although several works related to authentication and key agreement protocols have been proposed in the past, there is a significant overhead in both communication and computation. To overcome this burden, a Chebyshev chaotic map-driven anonymous authentication scheme with a key agreement scheme is proposed in this work. This work shows integrates a chaotic map with anonymous authentication, thereby balancing security and efficiency. The proposed scheme removes the bilinear pairing operations, thereby reducing the time required for cryptographic operations, which leads to a significant reduction in computation overhead. Moreover, Quantitative evaluation shows that the proposed scheme achieves a total execution time of only 0.9494ms representing a (49.7%−71%) reduction compared to existing schemes (1.8878-3.7756 ms) and a communication overhead of 264 bytes, which is (13.2%−57.4%) lower than most related works (84–620) bytes. Furthermore, the security analysis section addresses the secure nature of the proposed protocol in contrast to different types of security attacks. The efficiency of the proposed schema is validated against similar works based on the computation time of cryptographic operations using the Cygwin platform and is proven to be noteworthy.

## 1. Introduction

The Global Road Safety Report 2023, presented by the World Health Organization (WHO), disclosed that over 1.19 million lives are lost each year due to road traffic accidents [1]. According to the Ministry of Road Transport and Highways (MORTH) of India, road accidents have become a leading cause of death, particularly affecting individuals aged 5–29 years [1,2]. Recent studies highlight the urgent need for an intelligent or smart transport system to help reduce road accidents. In this regard, the development of Vehicular Ad-Hoc Networks (VANETs) has emerged as a promising solution for intelligent transport systems, enabling vehicles to communicate by exchanging messages. These messages give information about roadblocks, They tell about diversions and traffic updates. This real-time data helps other drivers drive better. VANETs have changed normal road systems into smart ones. They allow vehicles to talk to roadside units. Roadside units are called RSUs. An RSU works like a small cell network. It sends information to other vehicles or RSUs. When a vehicle shares information with an RSU it is called V2R communication. V2R stands for vehicle to roadside unit. When vehicles talk to each other it is called V2V communication. V2V stands for vehicle to vehicle.

VANETs have three main parts. First there are vehicles with onboard units. Onboard units are called OBUs. Second there are roadside units along the roads. Roadside units are called RSUs. Third there is a trusted authority. The trusted authority is the central manager for the whole VANET system. VANETs use dedicated short-range communication (DSRC). The DSRC is a type of wireless technology. It helps V2V and V2R communication work. Table 1 represents DSRC channel configuration table uses the 5.9 GHz frequency band. It lets V2V and V2R share information. This information helps manage traffic. It also helps send alerts for emergency vehicles. Shows these channels are split into two groups. The first group has one control channel called CCH. The CCH sends safety messages. These messages include bad weather conditions. They include traffic updates. They also include accident alerts. The second group has six channels called service channels (SCH). These service channels send non-safety messages. Examples are Internet access and infotainment apps.

**Table 1:** DSRC Channels.

Channel Type	Priority	Frequency Range	Bandwidth	Purpose
CCH-178	High	5.885-5.895 GHz	10 MHz	Safety related messages
SCH-172	High	5.855-5.865 GHz	10 MHz	For highly communicating time-sensitive messages
SCH-174	Low-to-High	5.865-5.875 GHz	10 MHz	For Navigation assistance, traffic flow management
SCH-176	Low-to-High	5.875-5.885 GHz	10 MHz	For routing updates
SCH-180	Medium	5.895-5.905 GHz	10 MHz	For Vehicle safety messages
SCH-182	Low-to-Medium	5.905-5.915 GHz	10 MHz	Internet Access, navigation
SCH-184	Medium-to-High	5.915-5.925 GHz	10 MHz	High power applications like video streaming

Since VANET communication uses a public wireless network for V2V and V2R communications, it is essential to guarantee that all messages are properly communicated by authorized entities and that the integrity of the communicated messages is preserved and not lost during transmission. Therefore, a strong authentication protocol is crucial for VANETs to avoid serious security attacks and threats to the network. If a proper authentication mechanism is not implemented in VANETs, malicious entities may send bogus messages, such as false congestion reports and location information, which can lead to traffic flow manipulation. In VANETs, vehicle users exchange information about their locations and speeds with other vehicles. Without proper privacy preservation, attackers can track the location of vehicle users and their real identities, leading to the misuse of personal information. Therefore, it is necessary to protect the privacy of vehicle drivers from location tracking, identity profiling and eavesdropping. However, implementing a strong privacy-preserving mechanism can increase the computational cost and communication delay, thereby affecting the performance of VANETs in real time environments.

Hence, it is essential to prepare lightweight privacy-preserving mechanisms to maintain the communication cost and communication delay as low as possible. Where most existing works rely on heavy cryptographic computations such as elliptic curve cryptography (ECC) and Rivest–Shamir–Adleman (RSA), leading to higher computational latency and communication overhead. In connection to this, in this paper, a novel anonymous authentication schema is proposed with the support of Chebyshev polynomial. If these security measures are not addressed properly, it may lead to situations in which an unauthorized entity may send fake messages, such as fabricated traffic updates, which mislead the driving conditions of other drivers. If authorization is not addressed properly, an attacker can easily enter the VANET system and perform all illegal activities. Therefore, if VANETs do not follow strong security practices, they may be attacked by spoofing, message tampering and denial-of-service attacks. These attacks compromise the safety of VANET networks. This authorization plays an important role in VANETs; therefore, every genuine participating entity (vehicle, RSU and TA) in a VANET should follow a proper procedure. This procedure will ensure that only authorized participants share the information, stop impersonation of trusted sources and stop spreading fake messages by unauthorized parties. Maintaining the anonymity of participants in a VANET is as important as protecting their privacy. These preserve vehicles and other participating entities from disclosing their real identities and locations during communication. This anonymity will stop the tracking and profiling of vehicles and, finally, privacy breaches from occurring. If a privacy breach occurs, personal information, such as the original identity proof and current vehicle location, can be disclosed to the attacker. The authentication (one-way identity verification), mutual authentication (two-way verification) and key agreement (shared secret establishment). Therefore, security, authorization and anonymity are important concerns that must be addressed to maintain secure and privacy-preserving communications in VANETs.

Recently, the application of Chebyshev polynomials in cryptography has gained attention. Their use in key management for VANETs is particularly noteworthy. The unique mathematical properties of Chebyshev polynomials make them well-suited for this purpose. Properties such as the semigroup property and recurrence relations ensure the security of VANETs. While ECC performs well in static or semi-dynamic environments, its frequent re-authentication in VANETs is clumsier than evaluating Chebyshev polynomials, leading to delays. Similarly, lattice-based methods, with their significantly larger keys and messages compared to Chebyshev-based methods, result in high bandwidth and verification overhead. Since VANETs are highly sensitive to latency, the bulky operations of lattice methods are practically challenging for fast moving vehicles. In comparison to ECC and lattice approaches, Chebyshev polynomials offer the lightest, fastest and most practical solution. In VANETs, Chebyshev polynomials are employed to generate keys. The key agreement policy is crucial in VANET networks, providing authorizations for vehicles, RSUs and the TAs. For this purpose, the semigroup property of Chebyshev polynomials is utilized to generate public and secret keys for encryption and authorization. The security provided by the Chebyshev Polynomial Discrete Logarithm Problem (CPDLP) is considered sufficiently strong for various cryptographic operations because it can generate one-way functions in connection with chaotic maps. This combination strengthens the security. Chaotic maps can provide Pseudo-Random numbers. Randomness is an important factor for security in cryptography. Chaotic maps are unpredictable because even smaller changes in the initial values result in completely unpredictable results.

The primary contributions of this work are summarized as follows, addressing the conflicts and challenges:

- To propose a novel TA-based anonymous authentication protocol to secure the user’s identity while communicating with others.
- To propose a session key distribution protocol to preserve the confidentiality of the future communicating messages.

**Roadmap:** The remainder of this paper is organized as follows. Section 2 surveys the related work, highlighting the contributions and limitations of existing approaches. Section 3 outlines the preliminaries, including the system model, proofs of the semigroup basic property of Chebyshev polynomials, attack model and notation. Section 4 details the proposed model, followed by an extensive security analysis presented in Section 5. Section 6 presents the performance evaluation of the proposed scheme, its limitations. Section 7 elaborates on the analysis conducted via NS-3 simulations. Section 8 discusses the limitations of the current work and outlines directions for future research. Finally, Section 9 concludes the paper.

## 2. Related work

To provide appropriate security and privacy in VANETs, most research has focused primarily on developing methods for anonymous authentication and effective key management. Table 2 summarizes the existing authentication and key agreement schemes for VANETs. The table compares protocols based on key parameters such as cryptographic technique used, security features achieved (e.g., mutual authentication, privacy preservation, key agreement), computational and communication overhead. It also mentions the limitations. This survey highlights the research gaps that motivate our proposed scheme.

**Table 2:** Literature Survey.

S no	Author	Year	Contributions	Limitations	Testbed/Simulation Validation
1	Zhang et al. [3]	2021	Offers a PA-CRT method for VANETs that avoids long-term key storage and ensures fast, low-cost authentication.	Relies on trusted authorities and fingerprint checks; may not work well if attackers target multiple domains.	Simulation
2	Vasudev et al. [4]	2021	P2-SHARP is a lightweight, hash-based authentication protocol designed for secure and privacy-preserving warning message transmission in IoVs, addressing both local and global communication.	Depending on a centralized Trusted Authority and assumptions of secure initial registration, which may pose risks in practical deployments like scalability and hardware dependencies like TPDs remain insufficiently validated.	Simulation
3	Wang et al. [5]	2022	Proposed ISC-CPPA, a secure method that protects user privacy and allows easy access removal. It enables fast group verification.	The scheme needs a trusted authority and has not been tested in real-world conditions. This may face issues with key updates in large networks.	Simulation
4	Zhou et al. [6]	2023	Offers signature of knowledge (SoK) system for secure communication and smart contracts for public key management, with improved performance and lower cost.	The system was tested only in simulations, so real-world performance is unpredictable. It also needs improvements to handle high vehicle density and unstable network conditions.	Simulation
5	Sang et al. [7]	2023	PACM offers an authentication scheme for VANETs by integrating blockchain-based certificate management and lightweight cryptographic operations.	PACM depends on trusted entities and stable infrastructure, so it may not always be practical. It also faces challenges like blockchain latency and smart contract costs.	Simulation
6	Cheng et al. [8]	2023	Offers anonymous authentication mechanism and privacy-preserving reputation comparison algorithm for identifying reliable vehicles.	Fails to address vehicle reliability in dynamic networks.	Simulation
7	Cheng et al. [9]	2024	Offers group key distributions, anonymous identity authentication and minimizes signal overhead.	Did not cover potential threats like APT and insider threats.	Simulation
8	Yang et al. [10]	2024	Offers prevention of invalid single signatures and security under random oracle assumptions.	Failed to explain scalability issues when the number of single signatures increases.	Theoretical/Analytical
9	Baee et al. [11]	2024	Offers key updating protocol for long-term cryptography.	Lack of trust establishment and limited protocols for session management.	Simulation
10	Lam et al. [12]	2024	Offers trust evidence from RSU and uses path backtracking methods for identifying malicious activity.	Suffers from single point failure; if RSU fails, the complete system goes down.	Simulation
11	Tan et al. [13]	2023	Offers a secure key management system for VANETs that protects user privacy, supports flexible access and maintains accountability through encrypted logging.	Complex computations and reliance on trusted entities may affect performance and real-time use in busy or unpredictable environments.	Simulation
12	Lin et al. [14]	2023	Proposed EBCPA protocol using KeyDer, SoK and smart contracts to ensure secure, private and efficient authentication in VANETs.	High dependency on blockchain infrastructure; efficiency may still be constrained by blockchain latency.	Simulation
13	Liang et al. [15]	2023	Disclosed that the CLAS system is not strong enough against forgery attacks.	Did not address which attacks can be handled after algorithm enhancements.	Theoretical/Analytical
14	Bojjagani et al. [16]	2022	Improved CLAS scheme to fix forgery issues in Mei et al.’s design, enhancing security, privacy and efficiency.	The original scheme was vulnerable to forgery.	Theoretical/Analytical
15	Mansour et al. [17]	2021	Offers ALMS protocol using asymmetric keys with CRT to reduce processing and communication load while handling changing group members efficiently.	Scalability remains challenging in large-scale VANETs; asymmetric cryptography may incur higher key generation cost.	Simulation
16	Madani et al. [18]	2024	Offers Chebyshev polynomials on FPGA without using excess hardware and provides a simple key exchange system without third-party support.	Worked only on FPGA and did not explore ASIC setups. Major attack types were not addressed.	Testbed (FPGA Implementation)
17	Singh et al. [19]	2023	Offers a secure practice for IoT devices from different owners to communicate using Chebyshev polynomials and blockchain while avoiding central authorities.	The protocol is slow with large calculations and may not work well on low-power devices.	Simulation
18	Zang et al. [20]	2021	Details an energy-efficient authentication and key exchange scheme using improved Chebyshev polynomial algorithms for smart grids.	Does not address scalability or real-world deployment challenges such as infrastructure integration and high user concurrency.	Simulation
19	Mohamed et al. [21]	2025	Details smart and efficient batch authentication for EV grids using bilinear pairings and aggregate signatures to verify multiple EV signatures simultaneously.	Does not address handling dense networks and identifying malicious EVs.	Simulation
20	Wang et al. [22]	2025	Proposed CDAS and DDAS protocols for centralized and distributed electricity data management in V2G.	Fails to address real-world deployment and ignores dynamic pricing scenarios.	Simulation
21	Vangujar et al. [23]	2025	Proposed blockchain-based key management for V2G networks for batch processing with aggregated signatures that accommodate large EV groups.	Did not mention scalability challenges of blockchain in larger networks.	Simulation
22	Krishnan et al. [24]	2025	Offers SDN and MEC secure handover authentication scheme for 5G vehicular networks using handover keys to avoid reauthentication.	Does not address real-world implementations and practical blockchain challenges.	Simulation

To achieve guaranteed conditional privacy, Zang et al. [3] introduced PA-CRT, an authentication framework for VANET network based on the Chinese remainder theorem (CRT). It minimizes computational costs by using lightweight modulo operations and fingerprints for authentication, instead of long-term secret storage. This scheme provides traceability, anonymity and safeguards against replay and impersonation attacks on the user. To provide strong privacy and secure communication in IoVs, Vasudev et al. [4] introduced the P2-SHARP protocol, which mainly focuses on transmitting warning messages. To achieve resistance against replay, man-in-the-middle (MITM) and trusted platform device (TPD)-stolen attacks, the protocol is equipped with lightweight hash-based authentication.

Similarly, Wang et al. [5] suggested that there is a growing need for private and secure communication in VANETs. To address these challenges, he proposed the Improved security certificates conditional privacy preserving authentication (ISC-CPPA) method, which addresses challenges such as dynamic key updates, random tag encryption and certificate-less cryptography to improve privacy, traceability and performance. Previous methods, such as public key infrastructure (PKI) and IDC-based CPPA, encountered difficulties with escrow and key management (Li et al.). Despite efforts to increase security, Li et al.’s CL-CPPA method does not provide complete anonymity. Shim, Azees and Shen later proposed substitutes; however, they encountered challenges with attack resistance and revocation efficiency. Zhou et al. [6] proposed blockchain based smart contracts system for vehicular networks but failed to address regular scalability challenge of blockchain when it comes to practicality.

Privacy preservation maintains communication go smooth and secure in VANETs. The old traditional methods like PKI and digital signatures have problems. Sometimes it leads to single point failure. Sang et al. [7] created a lightweight secure model PACM (Phase Amplitude Coupling index). The PACM majorly uses ECC and XOR operations. It provides lightweight mutual authentication. By the help of bloom filters in PACM fast certificate verification can be performed. Cheng et al. [8] proposed the anonymous authentication and privacy preserving reliability evaluation (AARE) protocol. This protocol uses homomorphic encryption and mainly used for dense networks. These approaches increase the user privacy and confidentiality management. These cannot survive quantum attacks. Cheng et al. [9] introduced a new schema which is lightweight and quantum random number based. It uses zero knowledge proofs. This scheme guarantees strong security because of quantum random numbers. It also ensures scalability and low overhead.

Yang et al. [10] proposed Certificateless Aggregate Signature schemes (CLAS). They reduce overhead. They also remove key escrow issues. This work introduces a method that does not rely on pairing, a provably secure CLAS protocol featuring a new aggregation algorithm that supports public verification and highlights conditional privacy with traceability. Maintaining message integrity and privacy in VANETs is challenging because they are compromised by outdated or interdependent cryptographic keys. Therefore, due to this reason Baee et al. [11] described the importance of long-term key update mechanisms and the security risks generated through interdependent key structures. This work highlights the issues demanding a secure and efficient key update protocol, introducing FLKUP and FSKUP for long- and short-term key updates to ensure perfect, forward secrecy and unlinkability.

Lam et al. [12] highlights involvement of trust in VANETs is important. Few models use Bayesian theory, Some use Dempster-Shafer theory or DST. To handle ambiguity in better way. This work introduces trust management framework MEFPB. It handles different types of date i) direct data and ii) indirect data. MEFPB also boosts trust accuracy. It resists on-off attacks and black hole attacks and it resists path manipulation threats. It does this with a path-backtracking technique. Tan et al. [13] proposed an attribute-based authenticated key management system. It uses certificateless cryptography. It also addresses batch authentication. The system supports V2V anonymous and accountable communication.

The Lin et al. [14] But these methods are usually expensive. They may also need special trusted hardware. Some current blockchain systems like CertCoin and BPAS are slow. They are not very good at tracking. EBCPA solves these problems. It uses smart contracts, digital signatures and key generation methods. Liang et al. [15] proposed a Certificateless Aggregate Signature (CLAS). This method helps for safe vehicle communications. It uses certificates for this. This work finds a important problem in Mei’s work. Then it comes up with a better CLAS model. This new model is stronger. Which is capable to protect against Type I and Type II attacks. Bojjagani et al. [16] described a lightweight security protocol. It is called AKAP-IoV. It uses Elliptic Curve Cryptography or ECC. The protocol uses secure session key generation to support mutual authentication. Mansour et al. [17] proposed the ALMS protocol. This protocol addresses challenges such as multicast communication in dynamic vehicular environments. Existing solutions use asymmetric and symmetric key protocols with high computational costs. The ALMS protocol includes the Chinese remainder theorem (CRT), prime factorization and discrete algorithms to achieve efficient, scalable and secure key distribution with minimized overhead. Madani et al. [18] proposed Chebyshev polynomials algorithm designed to resist side channel attack with high-speed cryptographic operations with low resource used. However, the complexity of the side-channel countermeasure implementations may increase.

Singh et al. [19] described a framework that combines blockchain decentralization with Chebyshev polynomial cryptography for security and trust; however, this protocol suffers from challenges such as blockchain consensus mechanisms and scalability challenges. Zang et al. [20] proposed energy-efficient authentication and key agreements for smart grid environments with Chebyshev chaotic map’s cryptographic properties for secure key agreement. However, they failed to discuss the practical implementation challenges. Mohamed et al. [21] proposed a simple and efficient batch authentication scheme that can verify many electrical vehicles for Vehicle-to-Grid (V2G) networks at a time. It uses bilinear pairings and aggregate signatures to verify multiple electric vehicle (EV) signatures simultaneously, which helps save both time and computing resources. The schema also combines certificateless cryptography with blockchain to keep records secure and decentralized. While protecting user privacy through features like traceability and controlled identity sharing when required. The system works well in terms of speed, scalability and low cost when compared to traditional methods. But the schema leaves as it is does not look much into things like how blockchain affects storage for long periods and power utilizations. Wang et al. [22] proposed two procedures. i) CDAS (Centralized Data Authentication Scheme) tailored for centralized networks and improves encryption and signature verification and ii) DDAS (Distributed Data Authentication Scheme) targets distributed setups using a matrix structure to avoid system crashes and reduce communication overhead. Both methods showed better speed, security and efficiency than other approaches along with the privacy. However, this work fails to detail the implementation in real-world deployment. Vangujar et al. [23] developed a lightweight blockchain-based key management for V2G with batch processing by using aggregated signatures. This accommodates large groups of EVs to get authenticated and managed together, that results in reduced delays and communication needs. It maintains privacy and forward secrecy and uses smart contracts for key updates and revocations. Krishnan et al. [24] proposed a secure handover authentication scheme for 5G vehicular networks using SDN and MEC to reduce delay and overhead. However, this scheme uses a Handover Key (HK) for fast reauthentication without disturbing the base network. They proposed their future work with AI-based handover optimization and blockchain to achieve more security.

Current authentication and key agreement methods for VANETs have serious problems. These problems make them hard to use safely and efficiently. Real time scenarios change regularly. Majority methods depend on a CA or RSUs. But these can fail easily. These schemes influence to important attacks like Sybil or insider threats Our proposed scheme fills this gap. It uses chaotic maps as a fast and secure alternative. It provides anonymous authentication. It provides efficient key agreement. It gives better privacy. With low lower overhead. It also reduces dependence on CA.

## 3. Preliminaries

### 3.1 System model

**Fig 1:**
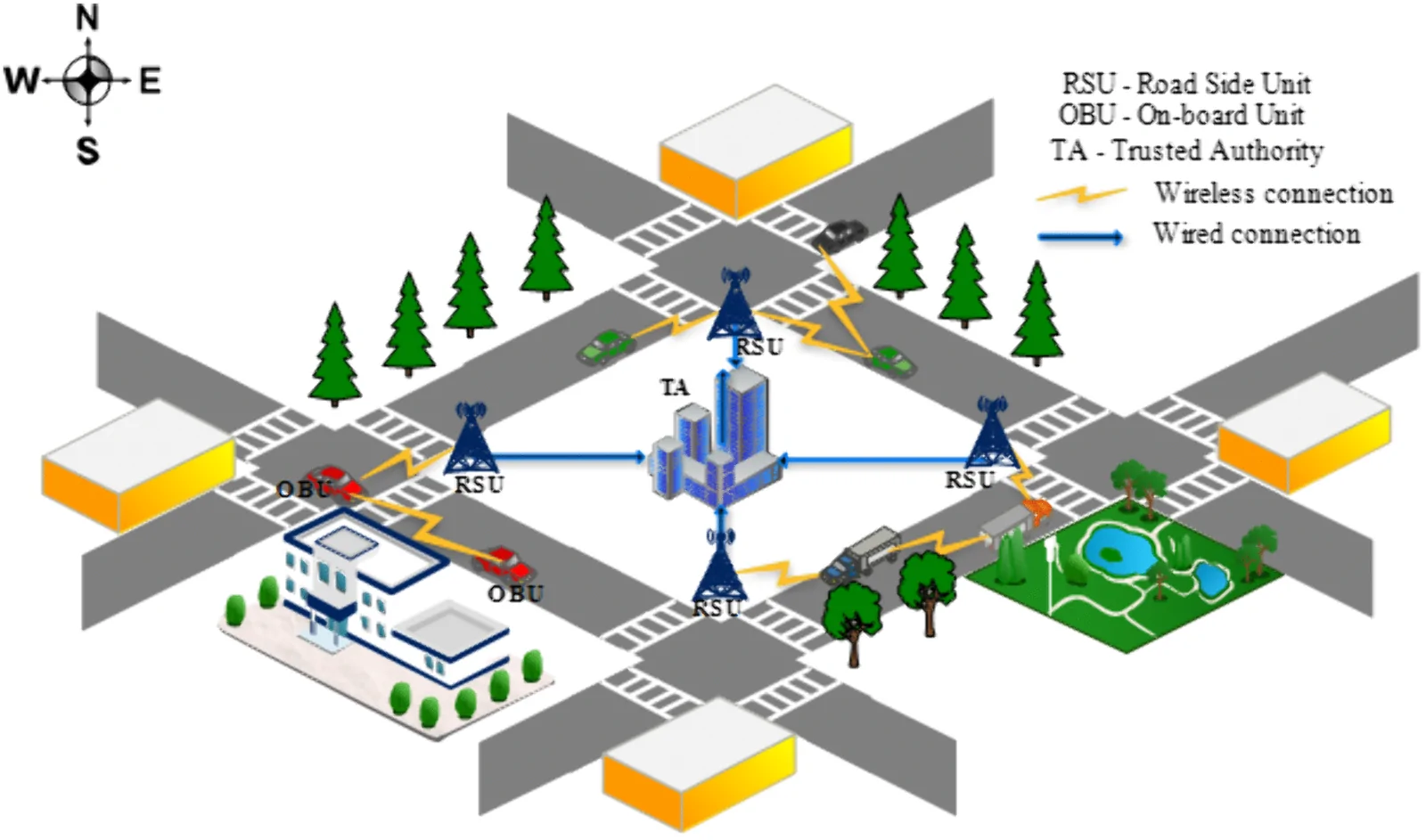
System architecture.

The system working architecture of the proposed schema is depicted in Fig 1. This architecture depicts real-time data transmission between vehicles, RSUs and TA. The main entities of the architecture are the TA, RSU and vehicle users.

**Trust Authority (TA)**: It acts as a centralized unit for the entire network. The major responsibilities of the TA include vehicle registration, RSU registration and secure credential distribution to the RSU and vehicles. The trusted authority is responsible for the registration of RSUs and vehicle users in the VANET system. During the registration period, the TA obtains the required credentials from the vehicle users and RSUs. Once the registration is completed, the TA issues some secret and public parameters to the vehicles and RSUs to make them authenticated entities of the VANET system. These issued parameters must be used by RSUs and vehicle users. Every geographic zone is assigned with a TA. However, the TAs of all geographic zones are interconnected with each other through fibre optic cables. Therefore, registration in any one TA is sufficient to prove its authenticity in the VANET system for vehicles or RSUs. Moreover, it is assumed that the TA has a strong firewall and intrusion detection system (IDS) to ensure that the TA is completely unbreakable because of security attacks.

**Vehicles**: In VANETs, vehicles serve as mobile nodes for data transmission and reception, integrated with On-Board Units (OBUs) that collect and validate traffic-related sensitive data, including position, velocity and traffic status. These vehicles share the information with other nearby vehicles as well as with the RSUs. Additionally, other units in the network provide useful services to vehicles, such as real-time updates and safety alerts. In general, inter-vehicle communication occurs every 100–300 milliseconds, enabling the rapid exchange of critical traffic data. This improves situational awareness and supports efficient decision-making in vehicles.

**Roadside Units (RSUs)**: These are deployed at roadside locations, RSUs serve as important interfaces between vehicles and other RSUs. RSUs gather and process data from authorized vehicles. After processing, they extract useful information from the data and transmit it to the TA and other vehicles. The TA stored this refined information for future reference. In later implementations, RSUs could be equipped with larger storage capacities; thus, due to this reason RSU can act as fog nodes for data storage to perform real-time information updates in large-scale networks.

### 3.2. Chebyshev polynomial

This section describes the fundamental characteristics of the Chebyshev polynomials and their computational challenges.

**Definition 1 (Chebyshev Polynomial Chaotic Map):** For any integers *n* and x∈[−1,1], the Chebyshev polynomial can be defined as either a trigonometric form which is the called cosine function (1) or recursive form which is represented in (2). In these the chaotic behaviour can be experienced in recursive form when n≥2 [25,26].


$$\begin{array}{c} T_{n}\left(x\right)=\cos\left(n\cos^{-1}\left(x\right)\right), \end{array}$$ (1)

$$\begin{array}{c} T_{n}\left(x\right)=2x\;T_{n-1}\left(x\right)-T_{n-2}\left(x\right);\;n\geq 2,\;T_{0}\left(x\right)=1,\;T_{1}\left(x\right)=x. \end{array}$$ (2)

**Definition 2 (Semigroup Property):** The semigroup property of the Chebyshev polynomial is defined by (3).


$$\begin{array}{c} T_{u}\left(T_{v}\left(x\right)\right)=T_{v}\left(T_{u}\left(x\right)\right)=T_{u\cdot v}\left(x\right). \end{array}$$ (3)

The Chebyshev polynomial semigroup property remains valid even when its domain is extended to the interval (−∞,+∞). The improved Chebyshev polynomial is defined in (4), where *p* represents a large prime number:


$$\begin{array}{c} T_{n}\left(x\right)=\left(2x\;T_{n-1}\left(x\right)-T_{n-2}\left(x\right)\right)\mathrm{mod}p,\;n\geq 2,\;x\in \left(-\infty ,+\infty \right) \end{array}$$ (4)

The Chebyshev polynomial possesses numerous significant properties; however, the semigroup property is particularly used in our proposed work. Table 3 represents Chebyshev polynomials $T_{n}(x)$ for degrees *n* = 1–11. These polynomials satisfy the recurrence relation $T_{n}(x)=2xT_{n-1}(x)-T_{n-2}(x)$ with initial values $T_{0}(x)=1$ and $T_{1}(x)=x$. For cryptographic applications, the domain is extended to x∈(−∞,+∞) and computations are performed modulo a large prime *p*, as shown in Equation (4). The chaotic nature of Chebyshev polynomials emerges for n≥2. Table 3 represents the Chebyshev polynomials of order up to *n* = 11.

**Table 3:** Chebyshev polynomials of different orders (*n*).

*n*	$T_{n}\left(x\right)$
*n* = 0	$T_{0}\left(x\right)=1$
*n* = 1	$T_{1}\left(x\right)=x$
*n* = 2	$T_{2}\left(x\right)=2x^{2}-1$
*n* = 3	$T_{3}\left(x\right)=4x^{3}-3x$
*n* = 4	$T_{4}\left(x\right)=8x^{4}-8x^{2}+1$
*n* = 5	$T_{5}\left(x\right)=16x^{5}-20x^{3}+5x$
*n* = 6	$T_{6}\left(x\right)=32x^{6}-48x^{4}+18x^{2}-1$
*n* = 7	$T_{7}\left(x\right)=64x^{7}-112x^{5}+56x^{3}-7x$
*n* = 8	$T_{8}\left(x\right)=128x^{8}-256x^{6}+160x^{4}-32x^{2}+1$
*n* = 9	$T_{9}\left(x\right)=256x^{9}-576x^{7}+432x^{5}-120x^{3}+9x$
*n* = 10	$T_{10}\left(x\right)=512x^{10}-1280x^{8}+1120x^{6}-400x^{4}+50x^{2}-1$
*n* = 11	$T_{11}\left(x\right)=1024x^{11}-2816x^{9}+2816x^{7}-1232x^{5}+220x^{3}-11x$

### 3.3. Proof of semigroup property

The proof of the semigroup property is described as follows by considering the values *u* = 2 and *v* = 3 from equation (3)


$$\begin{array}{c} T_{2}\left(x\right)=2x^{2}-1 \end{array}$$ (5)

$$\begin{array}{c} T_{3}\left(x\right)=4x^{3}-3x \end{array}$$ (6)

$$\begin{array}{cc} T_{2}\left(T_{3}\left(x\right)\right) & =T_{2}\left(4x^{3}-3x\right)\\ & =2{\left(4x^{3}-3x\right)}^{2}-1\\ & =2\left(16x^{6}-24x^{4}+9x^{2}\right)-1\\ & =32x^{6}-48x^{4}+18x^{2}-1 \end{array}$$ (7)

$$\begin{array}{cc} T_{3}\left(T_{2}\left(x\right)\right) & =T_{3}\left(2x^{2}-1\right)\\ & =4{\left(2x^{2}-1\right)}^{3}-3\left(2x^{2}-1\right)\\ & =4\left(8x^{6}-12x^{4}+6x^{2}-1\right)-3\left(2x^{2}-1\right)\\ & =32x^{6}-48x^{4}+24x^{2}-4-6x^{2}+3\\ & =32x^{6}-48x^{4}+18x^{2}-1 \end{array}$$ (8)

$$\begin{array}{c} T_{u\cdot v}\left(x\right)=T_{2\cdot 3}\left(x\right)=T_{6}\left(x\right) \end{array}$$ (9)

$$\begin{array}{cc} T_{6}\left(x\right) & =2x\cdot T_{5}\left(x\right)-T_{4}\left(x\right)\\ & =2x\left(16x^{5}-20x^{3}+5x\right)-\left(8x^{4}-8x^{2}+1\right)\\ & =32x^{6}-40x^{4}+10x^{2}-8x^{4}+8x^{2}-1\\ & =32x^{6}-48x^{4}+18x^{2}-1 \end{array}$$ (10)

### 3.4. Computational Hardness Assumptions

The security of the proposed scheme relies on the fact that certain mathematical problems related to extended Chebyshev polynomials over a finite field are very hard to solve.

**Definition 3 (Chebyshev Chaotic Map-based Discrete Logarithm Problem (CMDLP))**: Suppose we are given two values *x* and *y*, where:


$$\begin{array}{c} y=T_{r}(x)\;\mathrm{mod}\;p \end{array}$$ (11)

It is computationally very difficult for any probabilistic polynomial attacker (PPT) to find the value of *r*. In simple terms, if the attacker is successful in finding the values of *x* and $T_{r}(x)\;\mathrm{mod}\;p$, it is hard to determine *r*. For large prime numbers *p* and x∈(−∞,+∞), the probability that any PPT adversary *A* can find *r* is extremely small.


$$\begin{array}{c} {\text{Adv}}_{A}^{\text{CMDLP}}=\mathrm{Pr}[A(x,T_{r}(x)\;\mathrm{mod}\;p)=r]<\epsilon (\lambda ) \end{array}$$ (12)

where λ is a negligible function. Equation (11) specifies that the probability of any probabilistic polynomial time adversary *A* successfully finding the secret integer *r* is less than a negligible function ϵ(λ) [26].

**Definition 4 (Chebyshev Chaotic Map-based Diffie-Hellman Problem (CMCDHP))**: Now suppose we are given three values *x*, $T_{r}(x)\;\mathrm{mod}\;p$, $T_{s}(x)\;\mathrm{mod}\;p$ [25]. It is computationally very hard for a PPT attacker to compute equation (12) without knowing the values of *r* and *s*.


$$\begin{array}{c} T_{rs}(x)\;\mathrm{mod}\;p=T_{r}(T_{s}(x))\;\mathrm{mod}\;p=T_{s}(T_{r}(x))\;\mathrm{mod}\;p \end{array}$$ (13)

In simple terms, even if the intruder manages to have *x*, $T_{r}(x)$ and $T_{s}(x)$ values, they still cannot compute the shared value $T_{rs}(x)$ [25–27].

Formally, equation (13) specifies that the probability of any probabilistic polynomial time adversary *A* successfully finding the secret integer *r* is less than a negligible function ϵ(λ).


$$\begin{array}{c} {\text{Adv}}_{A}^{\text{CMCDHP}}=\mathrm{Pr}[A(x,T_{r}(x)\;\mathrm{mod}\;p,T_{s}(x)\;\mathrm{mod}\;p)=T_{rs}(x)\;\mathrm{mod}\;p]<\epsilon (\lambda ) \end{array}$$ (14)

### 3.5. Attack Model

In VANETs, the open and constantly changing communication environment presents special security challenges that make them vulnerable to various types of attacks. This work considers an adversary operating under the Dolev–Yao model [28], in which the attacker has full control over the communication channel. The adversary can intercept, modify, replay and inject messages into a network. The adversary has limited computing power. They cannot break secure cryptographic methods. These methods include encryption or signatures. Breaking them requires special keys. The attacker does not have those keys.

#### 3.5.1. Adversarial capabilities.

- **Network Control:** The adversary can fully control the communication channel. They can intercept messages. They can modify and inject new manipulated messages. They can resend the old messages. They can delete messages too.
- **Knowledge:** The adversary can know all protocols and algorithms. But they have no access to private cryptographic keys.
- **Computation:** The adversary is limited by standard computational hardness assumptions. So, they cannot break encryption and digital signatures.

#### 3.5.2. Attack scenarios.

The adversary may attempt the following attacks relevant to vehicular networks.

- **Bogus Information Attack:** The adversary attempts to inject false data for example fake traffic alerts to mislead vehicles and RSUs.
- **Impersonation Attack:** The adversary attempts to masquerade as a legitimate vehicle or RSU by spoofing valid identities.
- **RSU Replication Attack:** The adversary installs a rogue RSU or replicates a legitimate RSU identity to intercept or manipulate communication.
- **Denial-of-Service (DoS) Attack:** The adversary floods the channel with excessive malicious traffic to degrade service availability.

The possible attacks presented in Table 4 may occur on the VANET infrastructure. Therefore, due to this reason strong security features such as efficient authentication protocols, message integrity checks, encryption and privacy-preserving techniques are seriously suggested.

**Table 4:** Attack–Capability Mapping.

Attack Type	Active/Passive	Required Capability
Bogus Information	Active	Message injection
Impersonation	Active	Identity spoofing and message injection
RSU Replication	Active	Rogue RSU deployment and spoofed RSU credentials
Denial-of-Service (DoS)	Active	High-volume message flooding

### 3.6. Notations

Table 5 represents the list of notations and abbreviations to enhance the readability of the proposed work throughout all the sections.

**Table 5:** Notations.

Notations	Definitions
*b*	TA’s Private Key
*p*	Strong prime number
${PUB}_{TA}$	TA’s Public Key
*x*	Seed value, range {2, p−1}
*H*(.)	One-time secure hash function (SHA-256)
${ID}_{u_{i}}$	Real Identity of user $u_{i}$
$v_{i}$	Private key of user $u_{i}$
${PUB}_{u_{i}}$	Public key of user $u_{i}$
${DID}_{u_{i}}$	Dummy identity of user $u_{i}$
${ID}_{{RSU}_{i}}$	${RSU}_{i}$‘s Real identity
$r_{i}$	${RSU}_{i}$‘s private key
${PUB}_{{RSU}_{i}}$	${RSU}_{i}$‘s public key
${DID}_{{RSU}_{i}}$	Dummy identity of ${RSU}_{i}$
${TS}_{i}$	User’s Current timestamp
${TS}_{i+1}$	RSU’s Current timestamp
*TA*	Trusted Authority
$Z_{p}^{*}$	Multiplicative group mod *p*
ΔT	Allowed Time Window differences
*A*	Used in $\alpha_{i}$
*B*	Used in $\beta_{i}$
*C*	Used by TA to verify $\beta_{i}$
*D*	Used by TA to verify $\alpha_{i}$
$\alpha_{i}$	User Authenticator
$\beta_{i}$	RSU Authenticator
$E_{1},\;E_{2}$	Encapsulation hash for RSU and User
*m*,*n*	Random nonces (Chosen by TA for session key derivation)
*I*_1_	Authentication process initiated by $u_{i}$
*I*_2_	Authentication process initiated by RSU including with *I*_1_
$I_{3},\;I_{4}$	Masked values for *m*,*n*
${SK}_{i}$	Session Key
$I_{5},\;I_{6}$	Masked session key through via *m*,*n*
*I*_7_	Tuple transferred from TA to RSU
$E_{{SK}_{i}}$	Symmetric Encryption (Encrypts payload with ${SK}_{i}$)
$D_{{SK}_{i}}$	Symmetric Decryption (Decrypts ciphertext with ${SK}_{i}$)
${Adv}_{A}^{CMDLP}$	Success rate or advantage of an attacker in breaking the Chebyshev discrete logarithm problem
${Adv}_{A}^{CMCDHP}$	Advantage of adversary *A* in solving the Chebyshev chaotic map-based Diffie-Hellman problem (CMCDHP)

## 4. Proposed work

Initially, the TA chooses a random integer *x* generated within the range {2, p−1}, where *p* is a large prime number. Further, the TA chooses a random number $b\;\epsilon \;{\mathbb{Z}}_{p}^{*}$ as private key. Furthermore, the TA computes its public key as ${\text{PUB}}_{TA}=T_{b}\left(x\right)\text{ mod }p$. Finally, the TA publishes further ${\text{PUB}}_{TA},\;x,\;p,\;H\left(.\right)$ as public parameters. Here, H(.) is the one-way secure hash function (SHA-256), which computes hashes of critical protocol data, guaranteeing both data integrity and one-way irreversibility. By combining the dynamically generated seed with hashing, our system provides robust protection against message tampering and unauthorized access while maintaining computational efficiency.

### 4.1. Vehicle registration ($u_{i}$)

In our protocol, a vehicle user ($u_{i}$) must directly visit the TA for the initial registration process. At the time of the initial registration process, the TA collects all the necessary details and documents from $u_{i}$ and verifies them with the support of any government-based organizations. After document verification is completed, the TA first assigns a real identity $\left({\text{ID}}_{u_{i}}\right)$ for $u_{i}$. Moreover, the TA computes the public key of $u_{i}$ as ${\text{PUB}}_{u_{i}}=T_{v_{i}}\left(x\right)\text{ mod }p$, where $v_{i}\;\epsilon \;{\mathbb{Z}}_{p}^{*}$ is the private key for $u_{i}$. To preserve the privacy of $u_{i}$, the TA also computes a dummy identity $\left({\text{DID}}_{u_{i}}\right)$ for $u_{i}$ as ${\text{DID}}_{u_{i}}={\text{ID}}_{u_{i}}⨁\;H(v_{i}||t)$. Here, the mapping of the dummy identity to the real identity is performed only at the TA. Moreover, the TA stores $\left({\text{PUB}}_{u_{i}},{\text{ID}}_{u_{i}},{\text{DID}}_{u_{i}},v_{i}\right)$ in its database for future reference. At the end of the vehicle registration process, the TA returns $\left({\text{PUB}}_{u_{i}},{\text{ID}}_{u_{i}},{\text{DID}}_{u_{i}},v_{i}\right)$ to $u_{i}$ in a secure manner.

### 4.2. RSU registration ($RSU_{i}$)

Similar to the vehicle registration process, each ${\text{RSU}}_{i}$ is required to register with the TA before its deployment on the roadside locations. In connection to this, the TA first assigns a real identity $\left({\text{ID}}_{{\text{RSU}}_{i}}\right)$ for ${\text{RSU}}_{i}$. Then the TA computes the public key $\left({\text{PUB}}_{{\text{RSU}}_{i}}\right)$ for ${\text{RSU}}_{i}$ as ${\text{PUB}}_{{\text{RSU}}_{i}}=T_{r_{i}}\left(x\right)\text{ mod }p$. Here, $r_{i}\;\epsilon \;{\mathbb{Z}}_{p}^{*}$ is the private key of ${\text{RSU}}_{i}$. Moreover, to preserve the privacy of ${\text{RSU}}_{i}$, the TA generates a dummy identity ${\text{DID}}_{{\text{RSU}}_{i}}={\text{ID}}_{{\text{RSU}}_{i}}⨁H(r_{i}||b)$. Finally, the TA provides $\left({\text{DID}}_{{\text{RSU}}_{i}},{\text{PUB}}_{{\text{RSU}}_{i}},r_{i},{\text{ID}}_{{\text{RSU}}_{i}}\right)$ to ${\text{RSU}}_{i}$ in a secure manner for future communications. Besides, the TA maintains these parameters in its database.

**Fig 2:**
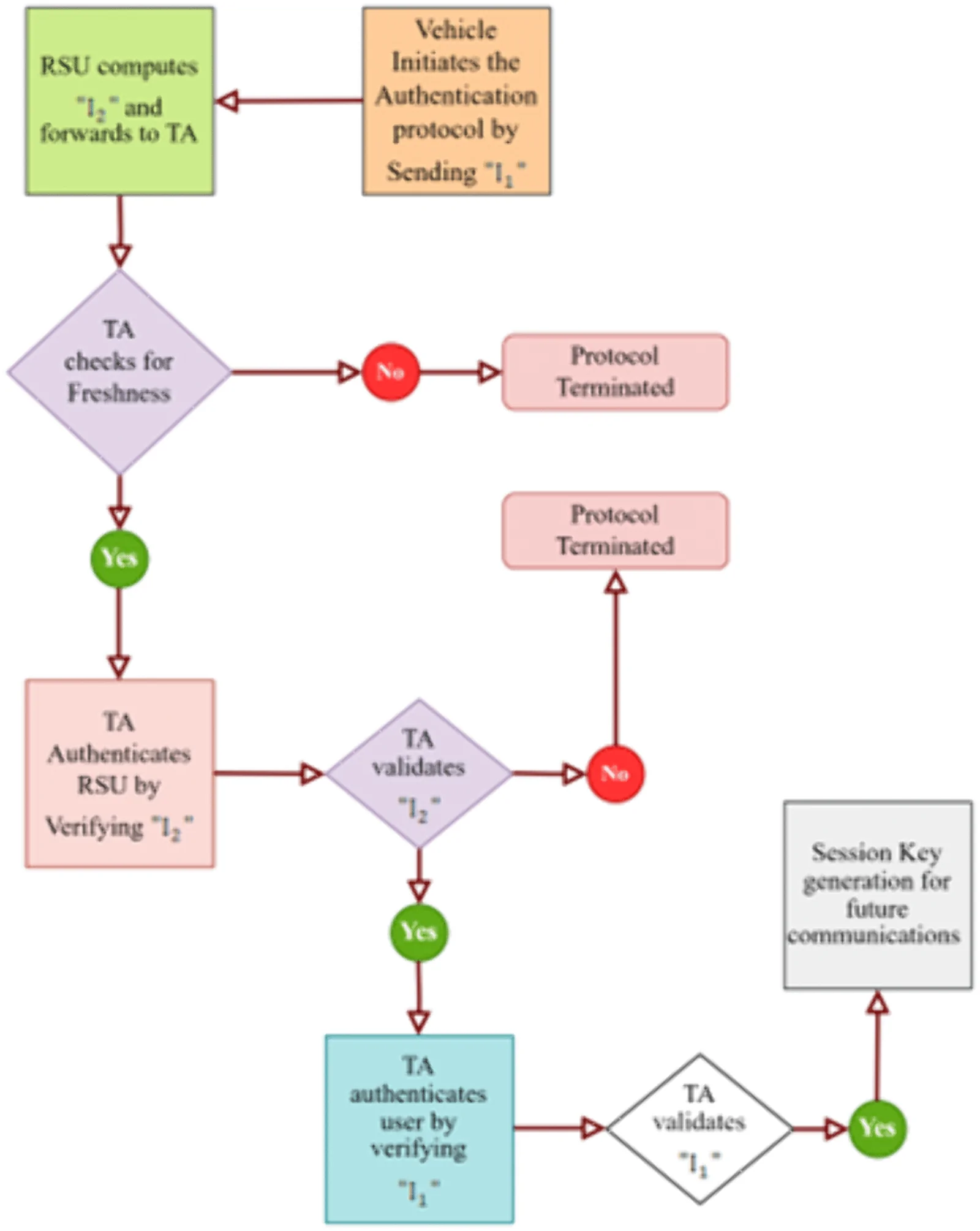
Anonymous authentication flowchart.

Fig 2 represents the flowchart of the anonymous authentication mechanism in proposed scheme between $u_{i}$, ${\text{RSU}}_{i}$ and TA.

- The authentication process is initiated by the user $\left(u_{i}\right)$, by sending *I*_1_ to the RSU $\left({\text{RSU}}_{i}\right)$ where *I*_1_ consists of all the relevant parameters of $u_{i}$.
- The ${\text{RSU}}_{i}$ then computes *I*_2_ and sends it to TA. The parameter *I*_2_ includes details of *I*_1_ too.
- The TA accepts *I*_2_ only if it passes the freshness test of timestamp $TS_{i+2}-TS_{i+1}=\Delta T$, if *I*_2_ fails to pass this test, the protocol will be terminated.
- If *I*_2_ is fresh, the TA validates the ${\text{RSU}}_{i}$ by checking whether $\beta_{i}^{*}=\beta_{i}$ or not. If this condition is satisfied, the TA authorizes the RSU; otherwise, the protocol is terminated.
- Once the TA authorizes RSU, it will authenticate the user by checking parameter *I*_1_, if the condition $\alpha_{i}^{*}=\alpha_{i}$ is satisfied the TA approves the user’s authentication, otherwise the protocol will be terminated.
- The TA generates session keys to perform secure communications between the RSU and user in the future.

The detailed explanation and execution of the anonymous authentication process are described in the following sections.

**Step 1**: To prove the anonymity to other entities in the network, $u_{i}$ must perform the following anonymous authentication process. In connection to this, $u_{i}$ first computes $\alpha_{i}$ in such a way that


$$\begin{array}{c} \alpha_{i}=H\left({\text{DID}}_{u_{i}}\left|\left|A\right|\right|TS_{i}\right) \end{array}$$ (15)

where,


$$\begin{array}{c} A=T_{v_{i}}\left(T_{b}\left(x\right)\right)\;\mathrm{mod}\;p, \end{array}$$ (16)

where:

- ${\text{DID}}_{u_{i}}$ is the dummy identity of the vehicle,
- $TS_{i}$ is the current timestamp to ensure message freshness,
- $A=T_{v_{i}}(T_{b}(x))\;\mathrm{mod}\;p$ is the chaotic-map-based shared value derived from the vehicle private key $v_{i}$ and the TA public parameter *b*,
- $TS_{i}$ represents the current timestamp.

Then, $u_{i}$ sends


$$\begin{array}{c} I_{1}=\left(\alpha_{i},{\text{DID}}_{u_{i}},TS_{i}\right)\text{ to }{\text{RSU}}_{i}. \end{array}$$ (17)

**Step 2**: By receiving *I*_1_ from $u_{i}$, the ${\text{RSU}}_{i}$ computes $\beta_{i}$ in such a way that


$$\begin{array}{c} \beta_{i}=H\left({\text{DID}}_{{\text{RSU}}_{i}}\left|\left|B\right|\right|TS_{i+1}\right) \end{array}$$ (18)

where,


$$\begin{array}{c} B=T_{r_{i}}\left(T_{b}\left(x\right)\right)\;\mathrm{mod}\;p \end{array}$$ (19)

then ${\text{RSU}}_{i}$ sends equation (19) to TA


$$\begin{array}{c} I_{2}=\left(I_{1},\beta_{i},{\text{DID}}_{{\text{RSU}}_{i}},TS_{i+1}\right)\text{ to the TA}. \end{array}$$ (20)

where:

- ${\text{DID}}_{{\text{RSU}}_{i}}$ is the RSU’s dummy identity,
- $TS_{i+1}$ is the RSU’s current timestamp and
- $B=T_{r_{i}}(T_{b}(x))\;\mathrm{mod}\;p$ is the RSU’s ephemeral chaotic value generated using its random nonce $r_{i}$,
- $TS_{i+1}$ represents the current timestamp.

Step 3: By receiving *I*_2_, the TA first checks whether $TS_{i+2}-TS_{i+1}\leq \Delta T$. If this condition is satisfied, then *I*_2_ will be accepted, otherwise, it will be rejected. Once the timestamp verification is successfully completed, the TA computes


$$\begin{array}{c} \beta_{i}^{*}=H\left({\text{DID}}_{{\text{RSU}}_{i}}\left|\left|C\right|\right|TS_{i+1}\right) \end{array}$$ (21)

where $C=T_{b}\left(T_{r_{i}}\left(x\right)\right)$, is the TA-computed verification value corresponding to the RSU’s nonce-based chaotic calculation.

Then, the TA checks whether $\beta_{i}^{*}=\beta_{i}$, if it is correct, then the TA extracts *I*_1_ from *I*_2_ and checks the authenticity of $u_{i}$ by ensuring


$$\begin{array}{c} \alpha_{i}^{*}=H\left({\text{DID}}_{u_{i}}\left|\left|D\right|\right|TS_{i}\right) \end{array}$$ (22)

where $D=T_{b}\left(T_{v_{i}}\left(x\right)\right)$, is the TA’s verification value for the vehicle’s chaotic-map authentication. If $\alpha_{i}^{*}=\alpha_{i}$, the vehicle $u_{i}$ is authenticated.

**Step 4**: TA computes the following parameters

The TA computes two masking values:


$$\begin{array}{c} E_{1}=H\left({\text{PUB}}_{{\text{RSU}}_{i}}\left|\left|r_{i}\right|\right|TS_{i+1}\right) \end{array}$$ (23)

$$\begin{array}{c} E_{2}=H\left({\text{PUB}}_{U_{i}}\left|\left|v_{i}\right|\right|TS_{i}\right) \end{array}$$ (24)

where:

- ${\text{PUB}}_{{\text{RSU}}_{i}}$ and ${\text{PUB}}_{u_{i}}$ are public keys of RSU and vehicle respectively,
- $r_{i}$ and $v_{i}$ are their private random secrets.

$$\begin{array}{c} I_{3}=H\left(m\right)⨁E_{1},\text{ where }m\in {\mathbb{Z}}_{p}^{*} \end{array}$$ (25)

$$\begin{array}{c} I_{4}=H\left(n\right)⨁E_{2},\text{ where }n\in {\mathbb{Z}}_{p}^{*} \end{array}$$ (26)

Where *m*,*n* are random nonces chosen by TA.

**Session Key**:


$$\begin{array}{c} {\text{Sk}}_{i}=H\left(m\left|\left|n\right|\right|r_{i}||v_{i}\right) \end{array}$$ (27)

$$\begin{array}{c} I_{5}={\text{Sk}}_{i}⊕H\left(m\right) \end{array}$$ (28)

$$\begin{array}{c} I_{6}={\text{Sk}}_{i}⊕H\left(n\right) \end{array}$$ (29)

Finally, TA sends equation (29) to ${\text{RSU}}_{i}$


$$\begin{array}{c} I_{7}=\left(I_{3},I_{4},I_{5},I_{6},TS_{i+3}\right) \end{array}$$ (30)

Then ${\text{RSU}}_{i}$ calculates *E*_1_ by using its own parameters along with $TS_{i+3}$, which is received from *I*_7_, to retrieve H(m). After that, ${\text{RSU}}_{i}$ retrieves ${\text{Sk}}_{i}$ from *I*_5_ using H(m). Then, ${\text{RSU}}_{i}$ sends $\left\{I_{4},I_{6},TS_{i+4}\right\}$ to $u_{i}$. Upon receiving it, $u_{i}$ computes *E*_2_, retrieves H(n) from *I*_4_ and extracts ${\text{Sk}}_{i}$ from *I*_6_.

**Step 5**: By receiving *I*_7_ from TA, ${\text{RSU}}_{i}$ extracts H(m) from *I*_3_ as follows


$$\begin{array}{c} H\left(m\right)=I_{3}⨁E_{1}. \end{array}$$ (31)

Here, *E*_1_ can be calculated by ${\text{RSU}}_{i}$ using the parameters ${\text{PUB}}_{{\text{RSU}}_{i}}$, $r_{i}$, $TS_{i+1}$. After that, ${\text{RSU}}_{i}$ computes the session key ${\text{Sk}}_{i}$ from *I*_5_ in such a way that


$$\begin{array}{c} {\text{Sk}}_{i}=I_{5}⨁H\left(m\right). \end{array}$$ (32)

Then the ${\text{RSU}}_{i}$ sends $\left\{I_{4},I_{6},TS_{i+4}\right\}$ to $u_{i}$.

**Step 6**: By receiving $\left\{I_{4},I_{6},TS_{i+4}\right\}$, $u_{i}$ extracts H(n) from *I*_4_ by performing


$$\begin{array}{c} H\left(n\right)=I_{4}⨁E_{2}. \end{array}$$ (33)

Here *E*_2_ is calculated with the support of his private key $v_{i}$. After that, $u_{i}$ computes the session key ${\text{Sk}}_{i}$ from *I*_6_ in such way that


$$\begin{array}{c} {\text{Sk}}_{i}=I_{6}⨁H\left(n\right). \end{array}$$ (34)

Both parties now share the same session key ${\text{SK}}_{i}$.

**Fig 3:**
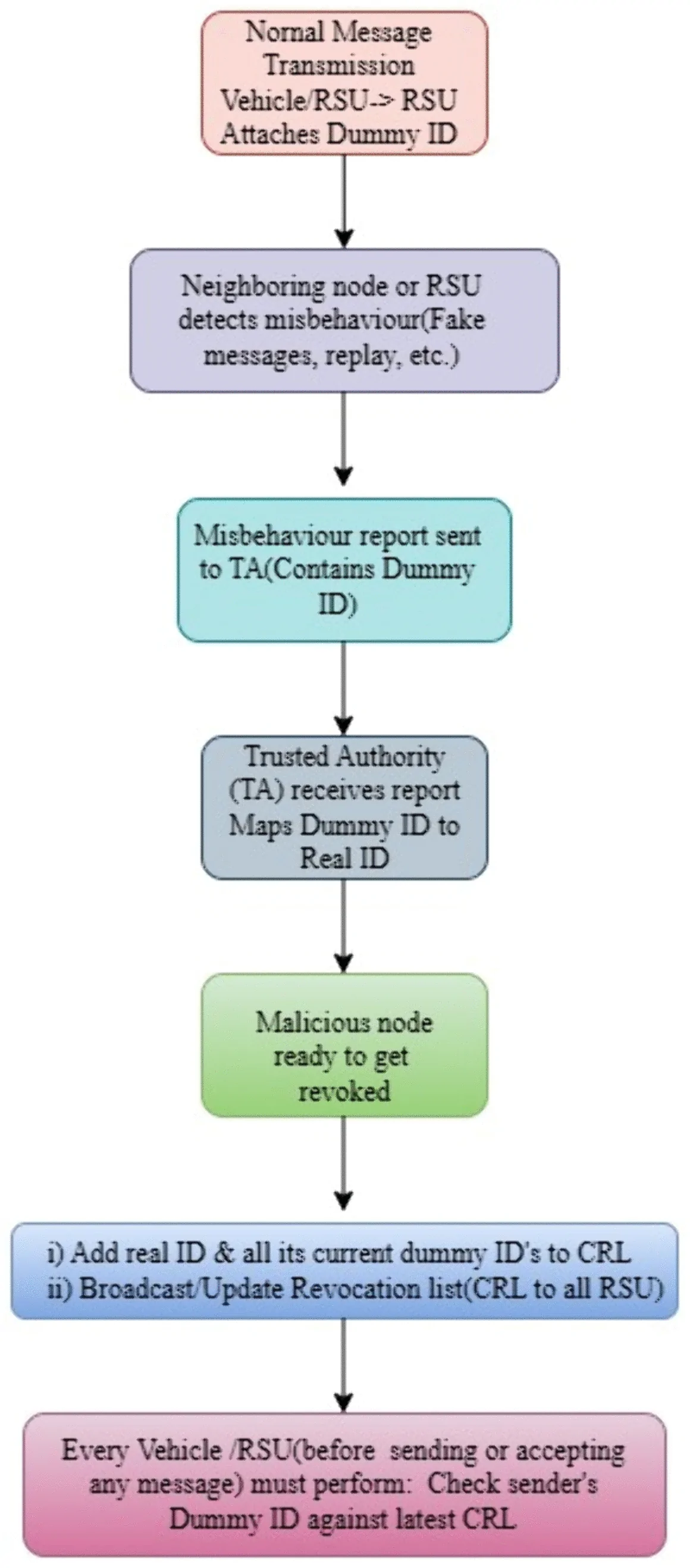
Revocation and identity tracing flowchart.

**Step 7**: During message transmission, vehicles and RSUs attach their dummy identities $\left({\text{DID}}_{u_{i}},{\text{DID}}_{{\text{RSU}}_{i}}\right)$ along with the messages. In the case of any misbehaviour, the TA can trace the vehicle or RSU using these dummy identities. After identifying the real identities $\left({\text{ID}}_{u_{i}},{\text{ID}}_{{\text{RSU}}_{i}}\right)$, the misbehaving vehicle or RSU is revoked from the VANET system and both their real and associated identities are added to the revocation list maintained by the TA. Before participating in communication, each vehicle or RSU must check the revocation list. Fig 3 illustrates the conditional privacy preserving revocation and identity tracing that when an identity appears on the list, the corresponding RSU or vehicle is denied permission to join the network.

After obtaining ${\text{Sk}}_{i}$, ${\text{Sk}}_{i}$ is used to make confidential communication with ${\text{RSU}}_{i}$ by the user. Here, ${\text{Sk}}_{i}$ is used as a symmetric key for a session established between the $u_{i}$ and ${\text{RSU}}_{i}$. Here, to preserve the confidentiality of message *m*, advanced symmetric key based encryption algorithms are used in this paper in support of ${\text{Sk}}_{i}$ as

Secure message transmission uses symmetric encryption:


$$\begin{array}{c} E_{{\text{SK}}_{i}}(m∥{\text{PUB}}_{u_{i}}∥{\text{PUB}}_{{\text{RSU}}_{i}}) \end{array}$$ (35)

and is decrypted by ${\text{RSU}}_{i}$:


$$\begin{array}{c} D_{{\text{SK}}_{i}}(E_{{\text{SK}}_{i}}(m∥{\text{PUB}}_{u_{i}}∥{\text{PUB}}_{{\text{RSU}}_{i}})) \end{array}$$ (36)

## 5. Security analysis

The following section explains how the proposed scheme addresses various security attacks through formal and informal analyses.

### 5.1. Formal security analysis

Formal security analysis based on the ROR model is explained in this section. The proposed Chebyshev polynomial based anonymous authentication protocol is analysed based on the ROR model [29]. The adversary *A* interacts with parties via Send, Execute, Reveal, Corrupt and a single Test query to a fresh session. Let b⟵{0,1} be the challenger’s hidden bit. Upon Test, the challenger returns the real session key if *b* = 0 and a random key of the same length if *b* = 1. The adversary outputs a guess $b^{\prime }$. The advantage of *A* in this experiment is


$$\begin{array}{c} {\text{Adv}}_{ROR}^{A}≜\epsilon =\left|\mathrm{Pr}\left[b^{\prime }=b\right]-\frac{1}{2}\right| \end{array}$$ (37)

Thus ϵ denotes the distinguishing advantage between a real session key and a random string.

#### 5.1.1. System Parameters.

- $q_{H}$: number of hash queries (modelled as RO of size $|\text{Hash}|=2^{h}$).
- $q_{s}$: bound on Send (active forgeries)/concurrent sessions relevant to a particular partner.
- *L*: session-key length (bits) output by the KDF.
- |*D*|: domain size of protocol nonces/challenges (timestamps/nonces/labels) needed to bind a forged message to a specific fresh session.
- Long-term secrets: vehicles $v_{i}$, RSU $r_{j}$. Session key ${\text{SK}}_{ij}$ depends on (i) Chebyshev-derived values and (ii) fresh nonces/challenges.
- Freshness: The Test session and its partner have not been revealed; neither both long-term keys $\left(v_{i},r_{j}\right)$ nor their session secrets are simultaneously exposed before Test.

#### 5.1.2. Game sequence and lemmas.

The games $G_{m}^{0}⟶G_{m}^{5}$ are used in this work and Succ(*G*) is used for the event where “*A* guesses *b* correctly in game *G*.” The bounding of ϵ will follow from a telescoping argument over these games.

**Game** $G_{m}^{0}$: **Real Protocol Lemma 1**.


$$\begin{array}{c} {\text{Adv}}^{A}(G_{m}^{0})=\left|2\mathrm{Pr}\left[\text{Succ}(G_{m}^{0})\right]-1\right|. \end{array}$$ (38)

*Explanation.*

**Game** $G_{m}^{1}$: **Passive Eavesdropping only** All executions are honest: *A* only observes $\left(\alpha_{i},{\text{DID}}_{u_{i}},TS_{i}\right)$, $\left(\beta_{j},{\text{DID}}_{{\text{RSU}}_{j}},TS_{i+1}\right)$.

**Lemma 2 (No passive gain):**


$$\begin{array}{c} \mathrm{Pr}\left[\text{Succ}(G_{m}^{1})\right]=\mathrm{Pr}\left[\text{Succ}(G_{m}^{0})\right] \end{array}$$ (39)

As a result, without $v_{i}$ and $r_{j}$, the key cannot be derived and there is no Test-distribution change.

**Game** $G_{m}^{2}$: **Active Send/Hash Forgeries** *A* crafts forgery. Authentication binds to RO outputs on transcripts containing $\alpha_{i},\beta_{j}$.

**Lemma 3 (Birthday bound):**


$$\begin{array}{c} \left|\mathrm{Pr}\left[\text{Succ}(G_{m}^{2})\right]-\mathrm{Pr}\left[\text{Succ}(G_{m}^{1})\right]\right|\leq \frac{q_{H}^{2}}{2|\text{Hash}|} \end{array}$$ (40)

As a result, any successful impersonation that changes distributions must induce a RO collision on some transcript component and apply birthday bound.

**Game** $G_{m}^{3}$: **Corrupt (Key Exposure)** *A* may corrupt either a vehicle or an RSU (but freshness forbids corrupting both partners before Test).

**Lemma 4 (Single-party exposure is insufficient):**


$$\begin{array}{c} \left|\mathrm{Pr}\left[\text{Succ}(G_{m}^{3})\right]-\mathrm{Pr}\left[\text{Succ}(G_{m}^{2})\right]\right|\leq \frac{q_{s}}{2^{L}|D|} \end{array}$$ (41)

As a result, with at most one long-term key being exposed, deriving ${\text{SK}}_{ij}$ still requires (a) binding to the correct fresh transcript (guessing a nonce/challenge with probability $\leq \frac{1}{\left|D\right|}$ per attempt) and (b) predicting the *L*-bit KDF output (probability $\leq 2^{-L}$). Over $q_{s}$ relevant attempts, union bound gives $\frac{q_{s}}{2^{L}|D|}$.

**Game** $G_{m}^{4}$: **Reduction to Chebyshev Hardness** If *A* can still distinguish, a solver *B* is built for the Chebyshev problem (i.e., recovering $T_{b}(x)$ or $T_{v_{i}}(T_{b}(x))$ without *b* or $v_{i}$) by embedding the challenge into a RO query/transcript.

**Lemma 5 (Reduction):**


$$\begin{array}{c} \left|\mathrm{Pr}\left[\text{Succ}(G_{m}^{4})\right]-\mathrm{Pr}\left[\text{Succ}(G_{m}^{3})\right]\right|\leq q_{H}\cdot {\text{Adv}}_{Chebyshev}(B). \end{array}$$ (42)

Standard RO-programming: *B* guesses the decisive RO query (probability $\leq \frac{1}{q_{H}}$) and answers using its Chebyshev challenge; success of *A* yields a solver for the hard problem.

**Game** $G_{m}^{5}$: **Final Guessing** All avenues closed; only random guessing remains.

**Lemma 6.**


$$\begin{array}{c} \mathrm{Pr}\left[\text{Succ}(G_{m}^{5})\right]=\frac{1}{2}. \end{array}$$ (43)

As a result, by construction, test key is independent random.

#### 5.1.3. Theorem: ROR Security of the proposed scheme.

**Theorem 1.** For any PPT adversary *A*, its advantage $\epsilon ={\text{Adv}}_{ROR}^{A}$ is bounded as


$$\begin{array}{c} \epsilon \leq \frac{q_{H}^{2}}{2|\text{Hash}|}+\frac{q_{s}}{2^{L}|D|}+q_{H}\cdot {\text{Adv}}_{Chebyshev}(B). \end{array}$$ (44)

*Proof:* Triangle inequality is applied over hybrids:


$$\begin{array}{c} \epsilon =\left|\mathrm{Pr}\left[\text{Succ}(G_{m}^{0})\right]-\frac{1}{2}\right|\leq \sum_{i=0}^{4}\left|\mathrm{Pr}\left[\text{Succ}(G_{m}^{i})\right]-\mathrm{Pr}\left[\text{Succ}(G_{m}^{i+1})\right]\right|. \end{array}$$ (45)

Bound each term by Lemmas 2–5; Lemma 6 gives the terminal $\frac{1}{2}$.

#### 5.1.4. Corollary: Adaptive indistinguishability with single-party corruption.

Let *A* be adaptive (may interleave Send/Execute/Reveal/Corrupt before/after session completion) and assume freshness (never both partners corrupted before Test and no Reveal of the Test session). Then the session key of the Test session is indistinguishable from random:


$$\begin{array}{c} \epsilon ={\text{Adv}}_{ROR}^{A}\leq \frac{q_{H}^{2}}{2|\text{Hash}|}+\frac{q_{s}}{2^{L}}+q_{H}\cdot {\text{Adv}}_{Chebyshev}(B)=\text{negl}(\lambda ). \end{array}$$ (46)

The adaptive setting does not change the Lemmas 3–5 under the freshness condition, and the single-party leakage is absorbed by Lemma 4’s term.

This expanded real-or-random (ROR) security model directly integrates the structure, keys and parameters of our proposed Chebyshev polynomial-based anonymous authentication protocol. Each security game matches one specific attack. The attack targets a step or a part of our scheme. This shows our scheme is strong. It can resist many types of attacks.

### 5.2. Formal security verification using scyther

**Fig 4:**
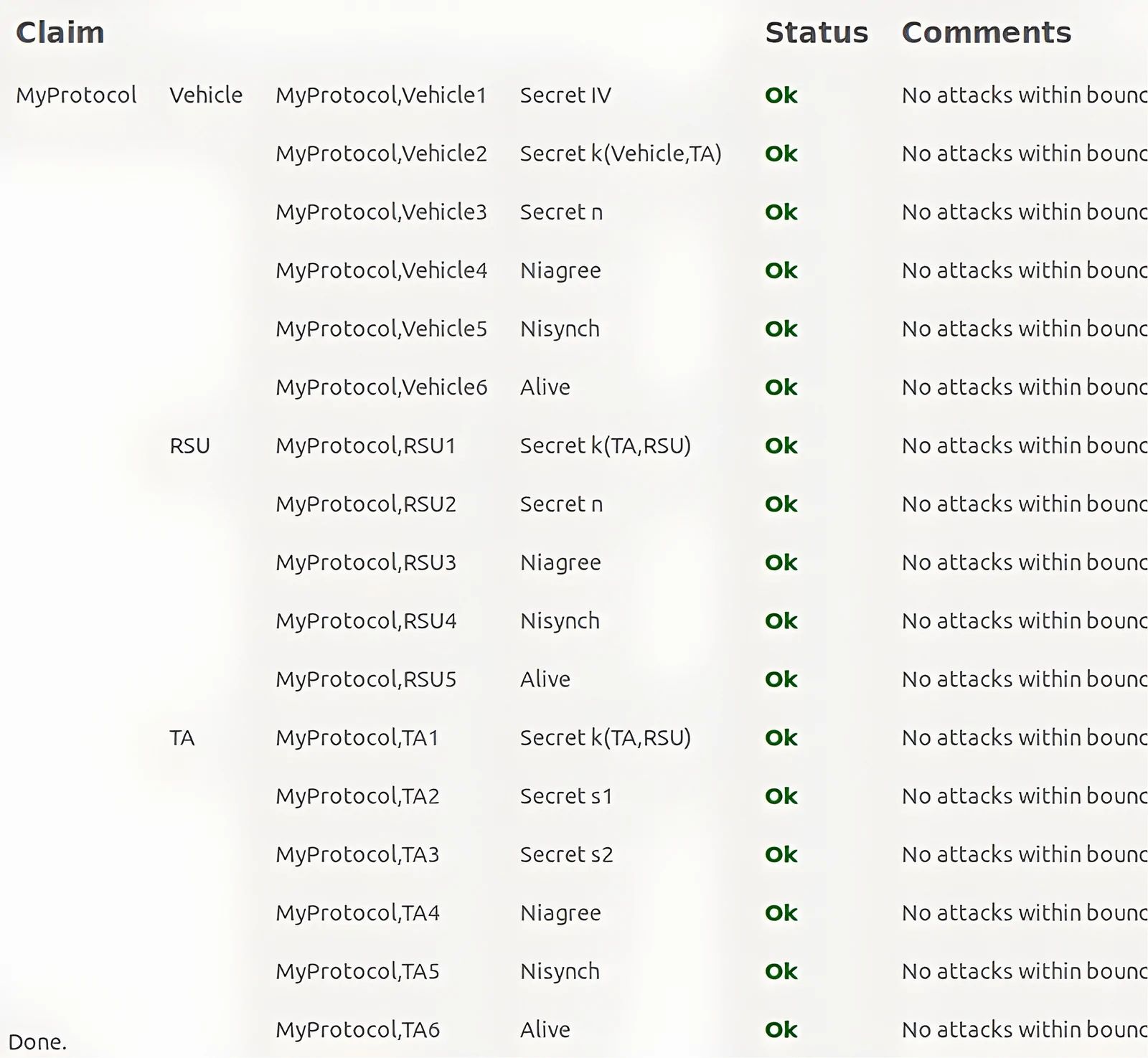
Formal security verification of the proposed work using Scyther.

The formal security analysis is essential for credibility. Accordingly, we have supplemented our informal security analysis with formal verification using the Scyther tool shown in Fig 4.

The results from the Scyther tool revealed the following observations:

- All secrecy claims (Secret IV, Secret k (vehicle, A), Secret n, Secret k (TA, RSU), Secret s1, Secret s2) were verified as OK-no attacks found.

*Description: All secrecy claims being “OK” means that under the Dolev-Yao attacker model (where the adversary controls the network), none of the secret values (temporary identifiers, shared keys, nonces, or master secrets) can be learned by the adversary. This formally proves the “confidentiality” and “privacy” of our protocol.*

- All liveness and synchronization claims (Niagree, Nisynch, Alive) for vehicles, RSUs and TAs were verified as ok-no attacks within bounds.

**Description:** All liveness and synchronization claims being “OK” means the protocol formally proves: (1) Mutual authentication is achieved between all parties (Vehicle ↔ RSU, RSU ↔ TA); (2) Replay attacks are impossible because nonce synchronization prevents message reuse; and (3) Protocol liveness is guaranteed because honest participants always complete the protocol successfully.

- The verification confirms that our protocol guarantees mutual authentication, session key secrecy and resistance to replay attacks under the Dolev-Yao attacker model.

Regarding public availability of Scyther code: While we fully support open science and reproducible research, the complete protocol specification contains proprietary implementation details that are currently part of a pending patent application / confidential institutional review. Therefore, we are unable to publicly release the full Scyther input files currently.

### 5.3. Informal security analysis

The Vehicle users and RSU signatures are important in this method because they help protect against security attacks. This method ensures that an outsider cannot create a valid user signature. The next section explains how this method protects against various attacks.

#### 5.3.1. Impersonation attack.

The proposed method withstands impersonation attacks. To execute an impersonation attack, the attacker $\left(\bar{A}\right)$ should pretend to be a user $\left(u_{i}\right)$ in the proposed work. However, $\bar{A}$ is unable to impersonate as $u_{i}$ due to the registration process in the TA. To impersonate as $u_{i}$, $\bar{A}$ has to first generate the value $\alpha_{i}$. To generate $\alpha_{i}$ the values ${\text{DID}}_{u_{i}}$ and *A* are required. However, ${\text{DID}}_{u_{i}}$ is not possible for $\bar{A}$ to be regenerated. To regenerate ${\text{DID}}_{u_{i}}$, $\bar{A}$ needs *b*. However, it is impossible for an adversary to get *b* from the ${\text{PUB}}_{TA}$. Since Chebyshev maps operate over finite fields, reversing ${\text{PUB}}_{TA}$ is very difficult and it is impossible for an adversary to calculate $\alpha_{i}$.

#### 5.3.2. Replay attack.

The proposed method counters replay attacks. In the proposed work, user sends $I_{1}=(\alpha_{i},{\text{DID}}_{u_{i}},TS_{i})$ to ${\text{RSU}}_{i}$. Once this message is received by ${\text{RSU}}_{i}$, it ensures the freshness of the timestamp $TS_{i}$ in such way that $TS_{j}-TS_{i}\leq \Delta T$. If this condition fails, the message *I*_1_ is simply rejected by the ${\text{RSU}}_{i}$. In this condition, $TS_{j}$ represents the time at which *I*_1_ is received by ${\text{RSU}}_{i}$ and ΔT represents the threshold value for time limit. Similarly, the TA also checks the timestamp for *I*_2_ by checking $TS_{j+1}-TS_{i+1}\leq \Delta T$. In our proposed work, the timestamp is attached to all the computational messages in such a way that our scheme can efficiently survive replay attacks.

#### 5.3.3. Message modification attack.

The proposed scheme is robust against message modification attacks. In our proposed scheme, $\alpha_{i}$ is calculated as $\alpha_{i}=H({\text{DID}}_{u_{i}}||A||TS_{i})$ where $A=T_{v_{i}}(T_{b}(x))\;\mathrm{mod}\;p$. To modify $\alpha_{i}$, the attacker has to know the value of $v_{i}$ from *A*. It is impossible due to the hardness of Chebyshev maps over finite field. Moreover, after sharing the session key ${\text{SK}}_{i}$ successfully, the message is encrypted using advanced symmetric encryption techniques. For instance, in the proposed work the message is encrypted using the session key


$$\begin{array}{c} M_{{\text{SK}}_{i}}(M||{\text{PUB}}_{u_{i}}||{\text{PUB}}_{{\text{RSU}}_{i}}) \end{array}$$ (47)

In this case, changing the message content *M* by breaking the symmetric encryption, such as 3-DES, within a polynomial time is highly impossible. Hence, our proposed scheme withstands message modification attacks.

#### 5.3.4. Privacy preservation.

The proposed scheme preserves the privacy of the user and RSU while communicating over the network. It is important to maintain the privacy of the user from attackers to prevent identity leakage. In connection to this, the proposed work includes dummy identities of the user and RSU during their communication in the channel. In the proposed work, $u_{i}$ transmits $I_{i}$ to ${\text{RSU}}_{i}$ and $I_{i}$ includes ${\text{DID}}_{u_{i}}$ to $u_{i}$. That is, instead of sharing the real identity, $u_{i}$ includes a dummy identity in such a way that the attacker cannot take any personal information about $u_{i}$. In the case of any dispute, the ${\text{DID}}_{u_{i}}$ of $u_{i}$ is mapped to the real identity of $u_{i}$ by the TA. Moreover, in the case of misbehaviour, the user is no longer authorized to access the system and their real identity is revealed to prevent further damage to the system. Similarly, the ${\text{RSU}}_{i}$ uses its dummy identity with others to preserve the system from identity-based attacks.

#### 5.3.5 Man-in-the-middle (MITM).

The proposed work withstands man-in-the-middle (MITM) attacks by ensuring that all communication between the user $u_{i}$, ${\text{RSU}}_{i}$ and the TA is protected using Chebyshev polynomial computations and timestamp-based freshness verification. In our scheme, the key value is an important component used to compute $\alpha_{i}=H({\text{DID}}_{u_{i}}||A||TS_{i})$. For an attacker $\bar{A}$ to successfully perform an MITM attack, they must regenerate $\alpha_{i}$ or the session key. However, since *A* is based on the private key $v_{i}$ and secure parameters from TA (like *b*) and due to the hardness of reversing the Chebyshev map over a finite field, it is computationally impossible for $\bar{A}$ to forge a valid *A*. Moreover, messages are secured using a symmetric encryption scheme with the session key $K_{i}$ and timestamps are validated to ensure freshness. Any tampering or delay introduced by $\bar{A}$ would be immediately detected and rejected by the system. Hence, the proposed scheme strongly resists MITM attacks.

### 5.4. Analysis against advanced threat models

Advanced threat models are essential for demonstrating the strength of any security protocol. The security analysis has been extended to explicitly address sophisticated threats and to clarify how the scheme is designed to mitigate insider threats and Sybil attacks.

#### 5.4.1. Strength against insider threats.

An insider threat involves an authorized, registered entity (e.g., a vehicle or RSU) turning malicious.

Threat Scenario: A registered vehicle $\left(u_{i}\right)$, with its valid credentials $\left(v_{i},{\text{PUB}}_{u_{i}},{\text{DID}}_{u_{i}}\right)$, starts behaving maliciously (e.g., sending false safety messages, attempting to track other vehicles).

- **Traceability:** The Trusted Authority (TA) can cryptographically trace the malicious activity back to the vehicle’s real identity ${\text{ID}}_{u_{i}}$. This is possible because the TA knows its master key *b* and can reverse the Dynamic ID operation:
  $$\begin{array}{c} {\text{ID}}_{u_{i}}={\text{DID}}_{u_{i}}⊕H(v_{i}∥b). \end{array}$$ (48)
- **Revocation:** Once identified, the TA can immediately revoke the vehicle by adding its public key ${\text{PUB}}_{u_{i}}$ to the Certificate Revocation List (CRL). This CRL is distributed to all RSUs.
- **Access Denial:** When an RSU receives an authentication attempt from ${\text{PUB}}_{u_{i}}$, it first checks the CRL. The RSU then denies access right away. It does not perform any cryptographic verification first. This makes the insider’s credentials useless.

Our system does not prevent an insider from becoming malicious but provides a rapid and effective mechanism to detect, trace and dismiss them from the network. This attempt can neutralize the threat.

#### 5.4.2. Strength against sybil attacks.

A Sybil attack works like this. One bad actor creates many fake identities. The goal is to overload the system or to gain too much influence.

Threat Scenario: An attacker attempts to register multiple vehicles with fake identities. The attacker wants to create a fake traffic jam or neutralize the authentication process.

- **Preregistration with TA:** Our protocol requires every vehicle to be uniquely registered with the central TA with government-issued ID proofs. This registration happens before the vehicle joins the network. This process is mandatory.
- **Identity Binding:** During registration, a real identity ${\text{ID}}_{u_{i}}$ is cryptographically linked to a specific public key ${\text{PUB}}_{u_{i}}$. The public key comes from the vehicle’s private key. The real identity is also linked to a trusted authority.
- **Cost of Identity:** Getting a valid identity has a high cost. The attacker must go through formal registration for each identity. So, the Sybil attack is stopped. An attacker cannot arbitrarily generate an infinite number of valid $\left(v_{i},{\text{PUB}}_{u_{i}},{\text{DID}}_{u_{i}}\right)$ tuples.

The central preregistration requirement blocks Sybil attacks. Our scheme guarantees that each authenticated identity corresponds to a single registered entity only.

## 6. Performance analysis

This section describes computational costs. It compares the proposed scheme with other existing schemes. The overall cost is measured by execution time; this time covers the authentication phase and verification phase for the User and the RSU.

### 6.1. Real-time feasibility assessment

To evaluate the practical applicability of the proposed VANET authentication scheme, the computational cost and communication overhead must satisfy the stringent latency requirements of real-time vehicular environments. For this purpose, the execution time of each cryptographic operation is evaluated individually to accurately estimate the overall computational cost of the authentication protocol. Table 6 presents the computational time required for various cryptographic operations, which serves as the basis for the subsequent computational cost analysis.

**Table 6:** Computational time for various Cryptographic operations.

Symbol	Description	Computational Time (ms)
$T_{cb}$	Time taken to perform Chebyshev polynomial operation	0.006929 ms
$T_{h}$	Time taken to perform Hashing (SHA-256)	0.001095 ms
$T_{x}$	Time taken to perform EX-OR operation	0.002363 ms
$T_{m}$	Time taken to perform Modulus operation	0.002281 ms
$T_{b}$	Time taken to perform Bilinear Pairing	0.158 ms
$T_{th}$	Time taken to perform one keyed hash function using chaos map	0.00344 ms
$T_{Esm}$	ECC scalar multiplication	1.093552 ms
$T_{Epa}$	ECC point addition	2.618347 ms
$T_{MoM}$	Modular Multiplication	0.000587 ms
$T_{MA(nxk)}$	Matrix Addition of order n×k	0.069246 ms
$T_{MM}$	Matrix Multiplication	6.002402 ms
$T_{sr}$	Sampling Random Vector	0.010069 ms
$T_{MVM}$	Matrix Vector Multiplication	0.057924 ms
$T_{VA}$	Matrix Vector Addition	0.000687 ms
$T_{VNM}$	Vector number Multiplication	0.000560 ms

These parameters ensure the proposed scheme is both secure and practical for deployment in VANET environments.

The measured operations include hash functions, Chebyshev polynomial evaluations, bilinear pairings, XOR, and modular operations. The protocol was implemented on a laptop with the following configuration: AMD Ryzen 5 7520U processor, Radeon graphics, 2.80 GHz CPU, 8GB RAM and Windows 11 Home operating system.

According to the proposed model, to perform the anonymous authentication process, the user $u_{i}$ and the ${\text{RSU}}_{i}$ together require two Chebyshev polynomial operations $\left(T_{cb}\right)$, two hash function operations $\left(T_{h}\right)$ and one keyed hash function $\left(T_{th}\right)$ using chaotic map. Similarly, for verification, the Trusted Authority (TA) needs two Chebyshev polynomial operations $\left(T_{cb}\right)$, two hash function operations $\left(T_{h}\right)$ and one keyed hash function $\left(T_{th}\right)$ using chaotic map. However, because these operations contribute a minimal computational cost, they were not considered in this performance analysis. The proposed protocol has been compared with other similar schemes to evaluate efficiency.

### 6.2. Computational cost

The computational efficiency of the proposed scheme was demonstrated in Table 7 that shows comparison with other existing schemes [30–33]. The proposed scheme considers two hashing operations and two Chebyshev polynomial computations at both the message generation and verification stages, resulting in $2T_{h}+2T_{cb}+T_{th}\approx 0.474734$ ms for each step and a total execution time of $4T_{h}+4T_{cb}+2T_{th}\approx 0.949468$ ms, which is significantly lower than all compared schemes [30–33] Specifically, when Compared to the existing schemes, the proposed work reduces the total computational cost by a minimum of 49.7% (against Yang et al. [30]) and a maximum of 74.9% (against Al-Shareeda et al. [33]). representing a substantial computational advantage. This improvement is attributed to the elimination of bilinear pairing operations and the use of efficient Chebyshev polynomial evaluations.

**Table 7:** Comparison of Computational cost with various schemes.

Schemes	Message generation	Message verification	Total execution time
Jiyun Yang et al. [30]	$2T_{h}+4T_{cb}+T_{th}=0.943908$ ms	$2T_{h}+4T_{cb}+T_{th}=0.943908$ ms	$4T_{h}+8T_{cb}+2T_{th}=1.887816$ ms
Jie Cui et al. [31]	$18T_{th}+7T_{cb}=1.661189$ ms	$13T_{th}+2T_{cb}=0.482954$ ms	$31T_{th}+9T_{c}=2.144143$ ms
Tomar et al. [32]	$8T_{h}+6T_{cb}=1.416002$ ms	$9T_{h}+10T_{cb}=2.35541$ ms	$17T_{h}+16T_{cb}=3.771412$ ms
Al-Shareeda et al. [33]	$2T_{h}+4T_{cb}+T_{th}=0.943908$ ms	3$(2T_{h}+4T_{cb}+T_{th})=2.831724$ ms	$8T_{h}+16T_{cb}+4T_{th}=3.775632$ ms
Proposed work	$2T_{h}+2T_{cb}+T_{th}=0.474734$ ms	$2T_{h}+2T_{cb}+T_{th}=0.474734$ ms	$4T_{h}+4T_{cb}+2T_{th}=0.949468$ ms

**Fig 5:**
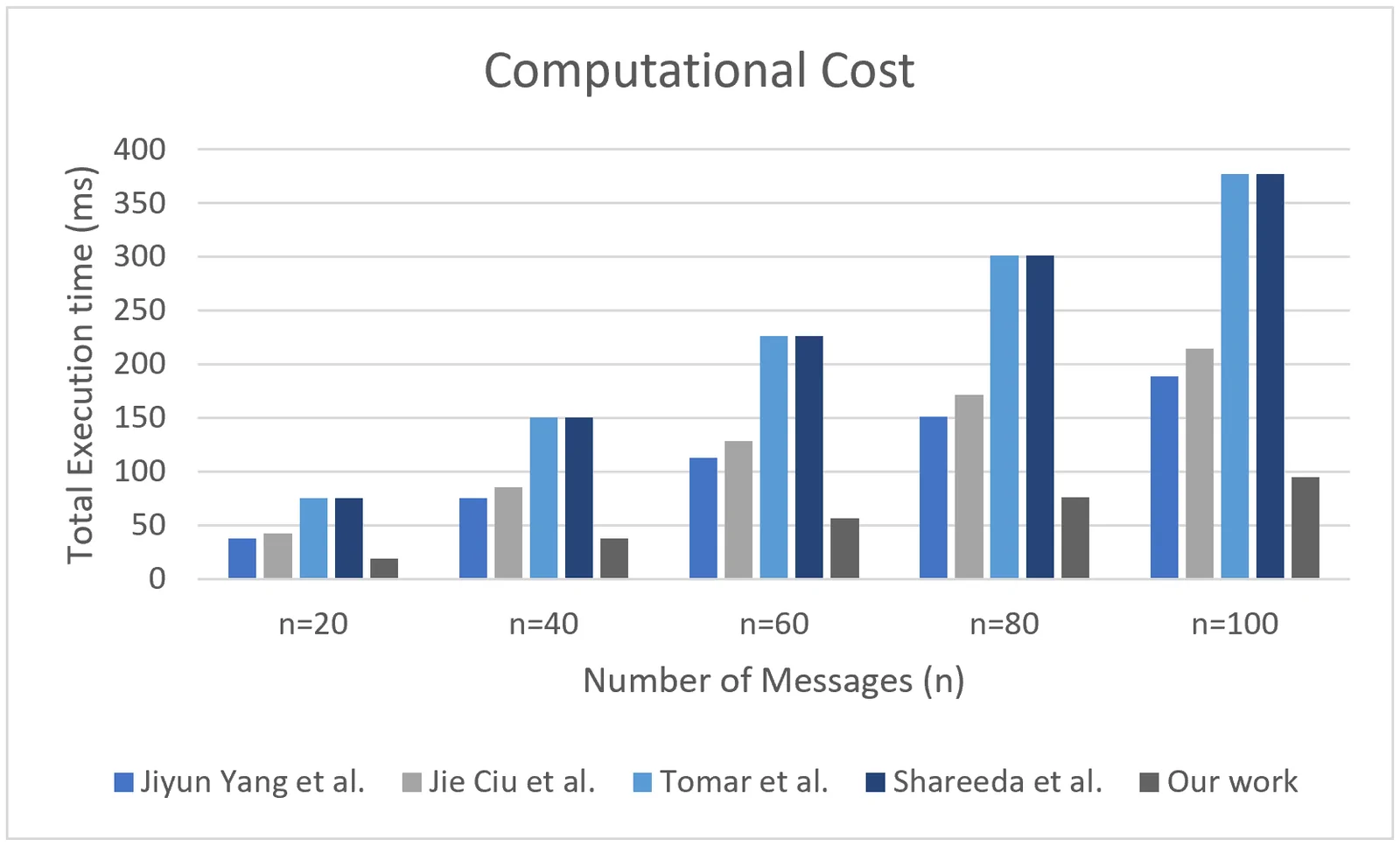
Comparison of computational cost (ms) for 1 to 100 vehicles with other schemes.

In comparison, Tomar et al. [32] has the highest computational cost, with a total execution time of $17T_{h}+16T_{cb}\approx 3.7714$ ms, due to the major reliability on multiple Chebyshev polynomial operations ($T_{cb}$) and hash function operations ($T_{h}$) across both message generation and verification phases. Jie Cui et al. [31] follows with a moderate total cost of $31T_{th}+9T_{cb}\approx 2.1441$ ms, primarily attributed to extensive point multiplication on ECC combined with Chebyshev polynomial operations ($T_{cb}$), which introduce significant modular exponentiation delays. Jiyun Yang et al. [30] shows a relatively lower total execution time of $4T_{h}+8T_{cb}+2T_{th}\approx 1.8878$ ms among the existing schemes, yet it still requires twice the number of Chebyshev polynomial operations compared to the proposed work. Whereas Shareeda et al. [33] evaluate the complete authentication process based on the execution time of cryptographic operations performed by the vehicle, Fog server, and Trusted Authority (TA). Specifically, the authentication phase comprises two hash function operations, four Chebyshev polynomial operations, and one chaotic map based keyed hash. The verification phase, in turn, incurs separate computational costs for the vehicle, Fog server, and TA, resulting in a total execution time that is approximately three times that of the authentication phase alone. shows the second highest computational cost of $8T_{h}+16T_{cb}+4T_{th}=3.775632$ ms due to extensive Chebyshev polynomial operations, hash operations, modular operations and multiple rotation functions. As shown in Fig 5, the bar graph represents total execution time (ms) for the proposed scheme with other existing schemes.

In contrast, the proposed scheme achieves the lowest computational overhead by far, requiring merely $4T_{h}+4T_{cb}+2T_{th}\approx 0.9495$ ms for the entire process, which is achieved by strategically eliminating redundant Chebyshev polynomial computations and optimizing the aggregate signature verification into a single streamlined step. Hence, The proposed scheme outperforms the others in terms of computational cost while ensuring security.

### 6.3. Communication cost

The communication cost analysis compares the proposed scheme with four related works [30–33], as shown in Table 8. In the proposed work, the Chebyshev polynomial computations, modular power operations and cryptographic hash algorithms each require 256 bits (32 bytes), whereas the current timestamp consumes 32 bits (4 bytes) of memory. Parameters transmitted from the User $\left(U_{i}\right)$ to the $\left({\text{RSU}}_{i}\right)$ include $I_{1}=(\alpha_{i},{\text{DID}}_{u_{i}},TS_{i})$, where $\alpha_{i}$ is computed as $\alpha_{i}=H({\text{DID}}_{u_{i}}||A||TS_{i})$, involving one Chebyshev polynomial computation, one hash operation and one timestamp, totalling 544 bits (68 bytes). Similarly, *I*_2_, transmitted from ${\text{RSU}}_{i}$ to the TA, also requires 544 bits (68 bytes). During TA verification, two sets of parameters $\left(E_{1},E_{2}\right)$ are needed to evaluate the ${\text{RSU}}_{i}$ and $V_{i}$, resulting in a total communication cost of 1024 bits (128 bytes), demonstrating the scheme’s efficiency in minimizing communication overhead while maintaining robust security.

**Table 8:** Comparison of communication overhead with other schemes.

Schemes	Communication cost
Jiyun Yang et al. [30]	84 bytes
Jie Cui et al. [31]	364 bytes
Tomar et al. [32]	620 bytes
Al Shareeda et al. [33]	304 bytes
Proposed work	264 bytes

In comparison, the complete authentication process according to Al Shareeda et al. [33] involves identity (20 bytes), the output of the Chebyshev polynomial (32 bytes), the hash value (20 bytes) and the timestamp (4 bytes). So, in total it comes around 76 bytes. The final communication cost includes Vehicle to Fog server, Fog server to Trusted Authority, Trusted Authority to Fog server and final Fog server to Vehicle. The final communication comes around 7*76=304 bytes. Tomar et al. [32] incur 236 bytes between Fog server and the vehicle. In addition, 384 bytes were used between the cloud and fog servers, summing to 620 bytes. Jie Cui et al. [31] requires 96 bytes for vehicle-to-TA communication, 96 bytes for UAV-to-TA, and 172 bytes for TA-to-UAV and TA-to-vehicle, so the total communication overhead needed is 364 bytes. Jiyun Yang et al. [30] achieves the lowest cost of 84 bytes by transmitting only these parameters $\left\{{\text{PID}}_{i},M,T_{i},MV\right\}$.

**Fig 6:**
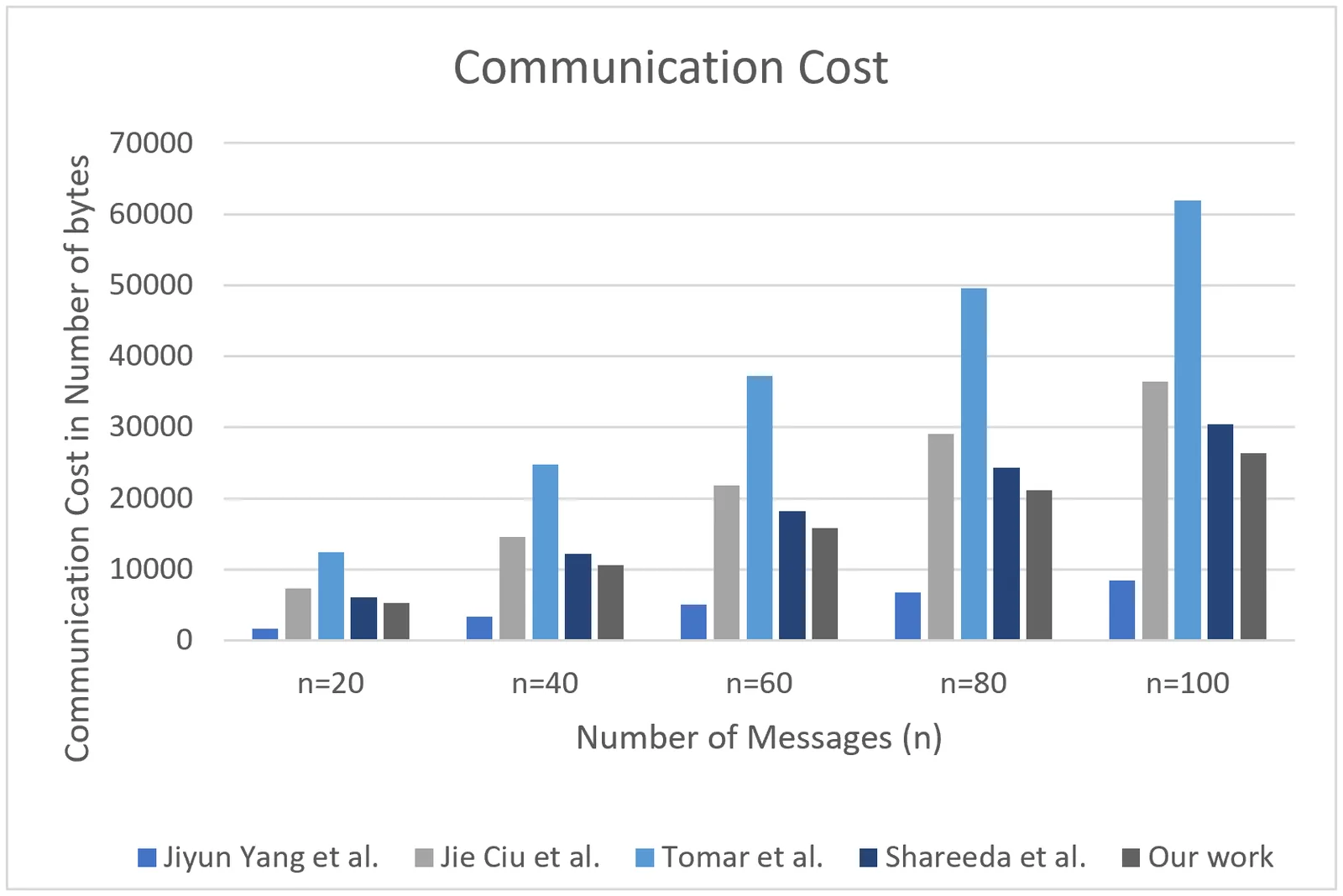
Comparison of communication overhead (bytes) for 1 to 100 vehicles with other schemes..

To accurately evaluate the communication efficiency of our proposed protocol, we compute the total end-to-end communication cost by analyzing the bit-length requirements of each transmitted message across the three sequential phases. As summarized in Fig 6, our scheme achieves a communication cost of 264 bytes. In our scheme, all cryptographic primitives including Chebyshev polynomial outputs, hash function outputs, and modular power operations are 256 bits (32 bytes) in size, while the timestamp is 32 bits (4 bytes).

The detailed breakdown is as follows:

- **Phase 1 (User to RSU):** The transmitted message is $I_{i}=(\alpha_{i},DID_{u_{i}},TS_{i})$, where $\alpha_{i}=H(DID_{u_{i}}∥A∥TS_{i})$ represents a hash output. This phase incurs a cost of Chebyshev polynomial (32 bytes) + Hash (32 bytes) + Timestamp (4 bytes) = **68 bytes**.
- **Phase 2 (RSU to TA):** The message *I*_2_ follows an identical structure to Phase 1, contributing another **68 bytes**.
- **Phase 3 (TA Verification):** The TA performs verification using two sets of parameters: $E_{1}=H(PUB_{RSU_{i}}∥r_{i}∥TS_{i+1})$ for the RSU, and $E_{2}=H(PUB_{U_{i}}∥v_{i}∥TS_{i})$ for the vehicle, where $r_{i}$ and $v_{i}$ are nonces of 32 bytes each. This phase collectively costs *E*_1_ (32 bytes) + $r_{i}$ (32 bytes) + *E*_2_ (32 bytes) + $v_{i}$ (32 bytes) = **128 bytes**.

Therefore, the total communication overhead of our proposed scheme is 68 + 68 + 128 = **264 bytes**.

As summarized in Table 8, our scheme achieves a communication cost of 264 bytes, which, while marginally higher than Jiyun Yang et al. [30] (84 bytes), significantly outperforms Jie Cui et al. [31] (364 bytes), Tomar et al. [32] (620 bytes), and Shareeda et al. [33] (304 bytes). This demonstrates that our protocol strikes an effective balance by minimizing communication overhead while maintaining robust security guarantees.

### 6.4. Storage overhead analysis

Storage overhead is an important performance metric for authentication protocols in VANETs, since On-Board Units (OBUs) and Roadside Units (RSUs) maintain very limited memory resources. Therefore, an efficient authentication protocol should minimize the amount of information stored by each participating entity while preserving the required security properties. This subsection analyzes the storage requirements of the proposed authentication protocol for the vehicle, RSU, and Trusted Authority (TA). To evaluate the storage analysis, the notations and parameter sizes considered in this work are summarized in Table 9.

**Table 9:** Notation and Size of Stated Parameters.

Parameters	Symbol	Size (bits)
Identity (Vehicle/RSU)	$i_{ID}$	160
Dummy Identity	$l_{DID}$	160
SHA-256 hash output	$l_{h}$	256
Chebyshev chaotic map parameter (private/public key)	$l_{c}$	160^*^
Session Key	$l_{SK}$	256
Timestamp	$l_{T}$	32

The size of the Chebyshev parameter depends on the selected prime modulus P. In this work, a 160-bit parameter is considered for the storage overhead analysis.

**Vehicle Storage:** After the registration phase, each vehicle stores its real identity ($ID_{u_{i}}$), dummy identity ($DID_{u_{i}}$), private key ($v_{i}$), and public key ($PUB_{u_{i}}$). Following a successful mutual authentication with the RSU, the vehicle additionally stores the negotiated session key ($SK_{i}$), which is used to secure subsequent communications. Therefore, the storage overhead of the vehicle can be expressed as:


$$\begin{array}{c} S_{OBU}={2l}_{ID}+2l_{c}+I_{SK} \end{array}$$ (49)

**RSU Storage:** Similarly, each RSU stores its real identity ($ID_{RSU_{i}}$), dummy identity ($DID_{RSU_{i}}$), private key ($r_{i}$), and public key ($pk_{RSU}$). After completing the authentication process, the RSU also stores the negotiated session key ($SK_{i}$) for secure communications with authenticated vehicles. Consequently, the storage overhead of the RSU is given by:


$$\begin{array}{c} S_{RSU}=2l_{ID}+2l_{c}+l_{SK} \end{array}$$ (50)

Since both the vehicle and the RSU maintain the same set of authentication credentials and session information, their storage requirements are identical.

**Trusted Authority Storage:** The Trusted Authority (TA) maintains its master private key (*b*) together with the registration database of all participating entities. For each registered vehicle, the TA stores the corresponding real identity, dummy identity, private key, and public key, namely the tuple $\left(ID_{u_{i}},DID_{u_{i}},v_{i},PUB_{u_{i}}\right)$. Likewise, for every registered RSU, the TA stores the tuple $\left(ID_{RSU_{i}},DID_{RSU_{i}},r_{i},PUB_{RSU_{i}}\right)$.

If the network contains *N* registered vehicles and *M* registered RSUs, the storage overhead at the TA is expressed as:


$$\begin{array}{c} S_{TA}=l_{c}+N(2l_{ID}+{2l}_{c})+M(2l_{ID}+2l_{c}) \end{array}$$ (51)

Using the parameter sizes listed in Table 9, the storage overhead of each entity is summarized in Table 10.

**Table 10:** Summary of Storage Overhead Per Entity.

Entity	Stored Parameters	Storage Formula	Storage (bits)
Vehicle (OBU)	$(ID_{u_{i}},DID_{u_{i}},v_{i},$ $PUB_{u_{i}},SK_{i})$	$2l_{ID}+2l_{c}+l_{SK}$	896
RSU	$(ID_{RSU_{i}},DID_{RSU_{i}},r_{i},$ $PUB_{RSU_{i}},SK_{i})$	$2l_{ID}+2l_{c}+l_{SK}$	896
TA (Master Key)	*b*	$l_{c}$	160
TA (per registered Vehicle)	$(ID_{u_{i}},DID_{u_{i}},v_{i},$ $PUB_{u_{i}})$	$2l_{ID}+2l_{c}$	640
TA (per registered RSU)	$(ID_{RSU_{i}},DID_{RSU_{i}},r_{i},$ $PUB_{RSU_{i}})$	$2l_{ID}+2l_{c}$	640

Therefore, the total storage requirement of the TA for a network containing N registered vehicles and M registered RSUs is


$$\begin{array}{c} S_{TA}=160+640N+640Mbits. \end{array}$$ (52)

The above analysis demonstrates that the proposed protocol imposes only 896 bits(112 bytes)of storage overhead on both the vehicle and the RSU, which is relatively small for modern vehicular hardware. The primary storage burden resides at the TA because it maintains the registrations database and identifies mapping for all participating entities. Since the TA is assumed to possess sufficient computational and storage resources, this centralized storage requirement does not affect the practicality or scalability of the proposed authentication protocol, Furthermore, the protocol does not require certificate repositories, verification tables, or additional authentication databases at either the vehicle or the RSU, making it well suited for resource constrained VANET environments

### 6.5. Performance comparison of chebyshev polynomials with ECC and Lattice-Based Models

To evaluate the efficiency of the proposed scheme, Table 11 compares the proposed Chebyshev polynomial-based scheme with existing works [34–37]. Zhu et al. [34] scheme incurs 12.231 ms due to scalar multiplications and pairings. Yang et al. [35] require 13.383 ms because of modular exponentiations, inversions and pairings. Liu et al. [36] present the most expensive scheme at 36.227 ms with complex multi-key and multi-matrix operations, unsuitable for latency-sensitive VANETs. Lil et al. [37] take 12.24 ms. This is moderate efficiency. They use matrix-vector operations. But this time is still higher than lightweight methods. Our proposed scheme is much faster. It completes authentication in just 0.949468 ms. It uses only hash functions and Chebyshev polynomial operations. This results in the lowest computational cost. So, our scheme proves that it is highly suitable for real-time VANET applications.

**Table 11:** Comparison of Chebyshev vs Non-Chebyshev Schemes.

Schemes	Message Generation + Message Verification	Total Execution Time
Zhu, D. et al. [34]	$\left(1\times T_{Esm}+1\times T_{H})+(3T_{Esm}+3T_{Epa}+2T_{H}\right)$	1.094+11.137=12.231 ms
Yang et al. [35]	$\left(T_{Esn}+3T_{Epa}+2T_{MoM}+T_{inverse}+2T_{H}\right)$ $+(4T_{Esm}+3T_{Epa}+3T_{H})$	1.151+12.232=13.383 ms
Liu et al. [36]	$\left(T_{H4}+T_{MA(nxk)}+T_{MM(mxm-mxk)}+T_{MM(mxk-kxk)}\right)$ $+(2T_{MA(nxk)}+2T_{M(nxk-kxk)}+$ $2T_{M(nxm-mxk)}+2T_{H0}+T_{H3}+T_{H4})$	12.075+24.152=36.227 ms
Lil et al. [37]	$\left(T_{sr}+T_{MVM}+2T_{NVM}+T_{H}+2T_{VA}\right)$ $+(T_{MVM}+2T_{NVM}+2T_{hash}+2T_{VA})$	6.016+6.008=12.024856 ms
Proposed work	$\left(2T_{h}+2T_{cb}+T_{th})+(2T_{h}+2T_{cb}+T_{th}\right)$	0.474734+0.474734=0.949468 ms

The proposed protocol offers three main advantages.

1. Our design never uses bilinear pairings. This act minimizes execution time.
2. We never use ECC operations; this act reduces scalar multiplications from three to two. That is a 33% reduction. This results in improved scalability.
3. We compare with lattice-based models [35,36]; it results in less communication cost when compared with others. It is practical for real deployment. It also works well in large and fast moving environments.

Table 6 represents the notations and descriptions of cryptographic and matrix operations. These parameters are used to calculate computational costs for existing schemes [34–37] in Table 11. It gives the notation details and the list of notations and their corresponding descriptions which are used in earlier sections.

**Fig 7:**
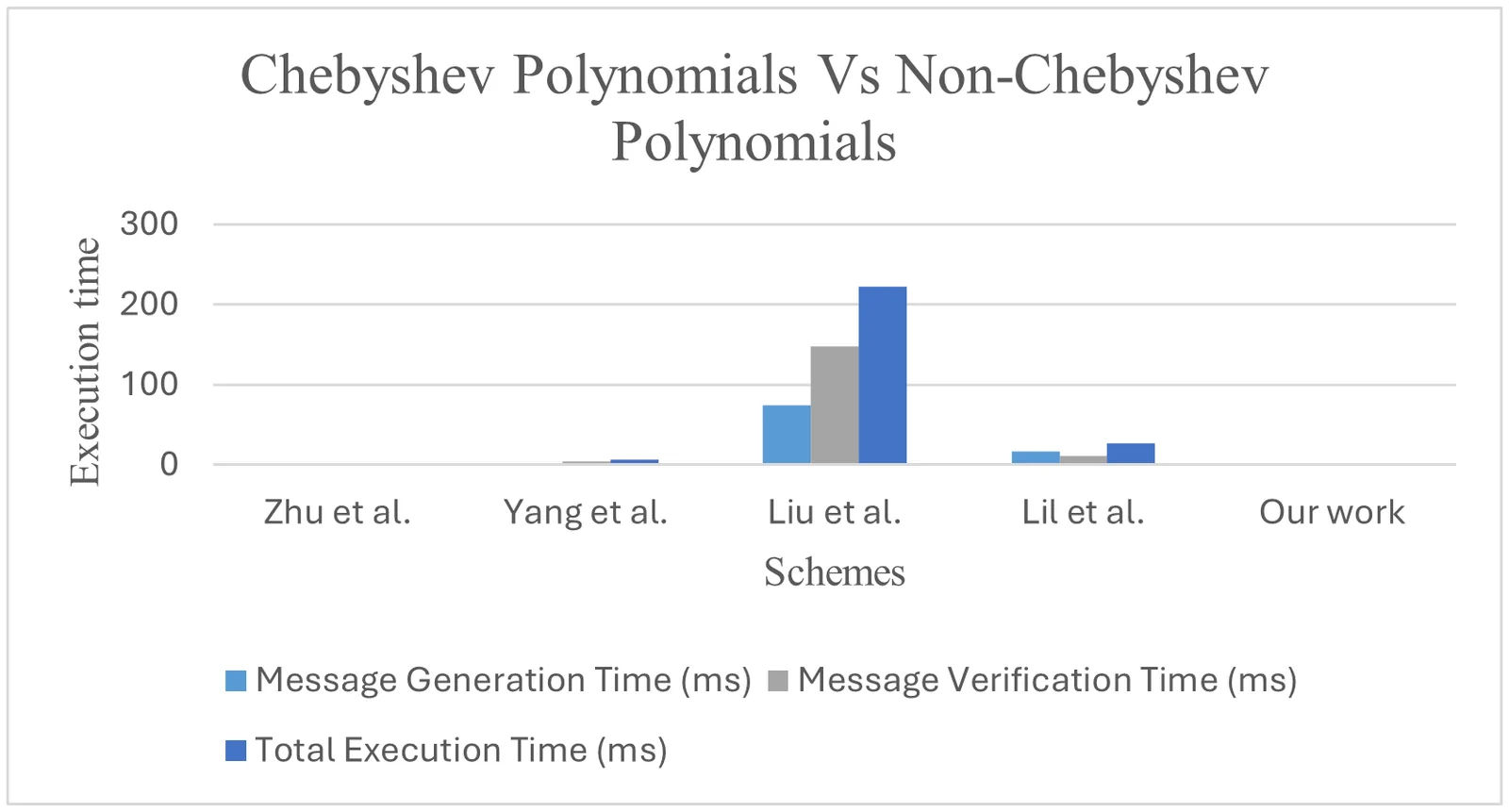
Comparative analysis of the proposed method versus ECC and lattice-based models.

Fig 7 represents a comparative bar graph of total execution time (in milliseconds) for the proposed Chebyshev-based scheme vs ECC-based and lattice-based cryptographic models. The proposed scheme outperforms both categories, achieving 0.949468 ms total cost. ECC-based schemes typically range from 10 to 15 ms, while lattice-based schemes [36, 37] require substantially higher overhead (12–37 ms) because of large matrix and vector operations. Our scheme achieves a remarkably low total execution time of just 0.949 ms, which is substantially lower than all competing schemes across both cryptographic paradigms. Specifically, when compared to the ECC-based schemes, our approach demonstrates a 99.20% improvement over Zhu et al. [36] (12.231 ms) and a 99.36% improvement over Yang et al. [37] (13.383 ms). The superiority of our scheme is even more pronounced against the lattice-based schemes, where we achieve a 98.95% improvement over Liu et al. [29] (36.227 ms) and an outstanding 99.99% improvement over Li et al. [28] (12.024 ms).

### 6.6. RSU Servicing Capability Analysis in VANET Environments

This section evaluates an RSU. The RSU can send signals up to 300 meters. This is a normal VANET with DSRC. We study two limits. One is how many authentications it can do. Two is the wireless channel. Table 12 lists the real-world system parameters for RSU servicing capability analysis in a DSRC-based VANET scenario.

**Table 12:** Parameters Used for RSU Servicing Capability Analysis in a DSRC-Based VANET Scenario.

Parameter	Symbol	Value	Description
RSU coverage radius	*R*	300 m	Typical DSRC range
Basic Safety Message (BSM) size	$S_{bsm}$	120 bytes	Includes safety data + security overhead
BSM transmission rate	$f_{bsm}$	10 Hz	Per SAE J2735
Channel bandwidth	*B*	10 MHz	Control Channel (CCH)
PHY (Physical layer) data rate	$R_{phy}$	6 Mbps	Robust mode (QPSK-½ OFDM)
Auth time per vehicle	$t_{auth}$	0.03209 ms	ECC signature processing
Maximum Number of Radio channels	$N_{radio}$	250-375	Depends on vehicle speed
Authentication limit	$N_{Auth}$	31,160 vehicles	Maximum number of vehicles authenticated by RSU
Area cell	$A_{cell}$	0.283 km^2^	Area cell will help in converting traffic density value into vehicle count

### 6.7. Key computations

**Bitrate Demand Per Vehicle**: Every vehicle transmits $f_{bsm}$ messages per second, each of size $S_{bsm}$:


$$\begin{array}{c} R_{veh}=S_{bsm}*8*f_{bsm}=120*8*10=9600\text{ bits/sec} \end{array}$$ (53)

**Channel Capacity with Efficiency**: Let η(v) be the channel efficiency, modelled as a function of speed *v* (km/h):


$$\begin{array}{c} \eta (v)=\left\{ \begin{array}{cc} 0.6, & \text{if }v<30\\ 0.5, & \text{if }30\leq v<60\\ 0.4, & \text{if }v\geq 60 \end{array}\right. \end{array}$$ (54)

Then the effective channel capacity is:


$$\begin{array}{c} C_{eff}=R_{phy}*\eta (v) \end{array}$$ (55)

$$\begin{array}{c} N_{radio}=\left\lfloor \frac{C_{eff}}{R_{veh}}\right\rfloor \end{array}$$ (56)

**Authentication Limit**: The number of authentications the RSU can perform per second is:


$$\begin{array}{c} N_{auth}=\left\lfloor \frac{1}{t_{auth}}\right\rfloor =\left\lfloor \frac{1}{0.03209*10^{-3}}\right\rfloor \approx 31,160 \end{array}$$ (57)

**Area**: For a coverage radius *R*, the area is:


$$\begin{array}{c} A_{cell}=\pi R^{2}=\pi *(0.3{)}^{2}\approx 0.283{\text{ km}}^{2} \end{array}$$ (58)

For traffic density ρ (vehicles/km^2^):


$$\begin{array}{c} N_{density}=\rho *A_{cell} \end{array}$$ (59)

**Final Servicing Capability**: The actual number of vehicles the RSU can support is:


$$\begin{array}{c} N_{served}=\min(N_{veh},N_{radio},N_{auth}) \end{array}$$ (60)

Where $N_{veh}$ is the number of vehicles present.

**Fig 8:**
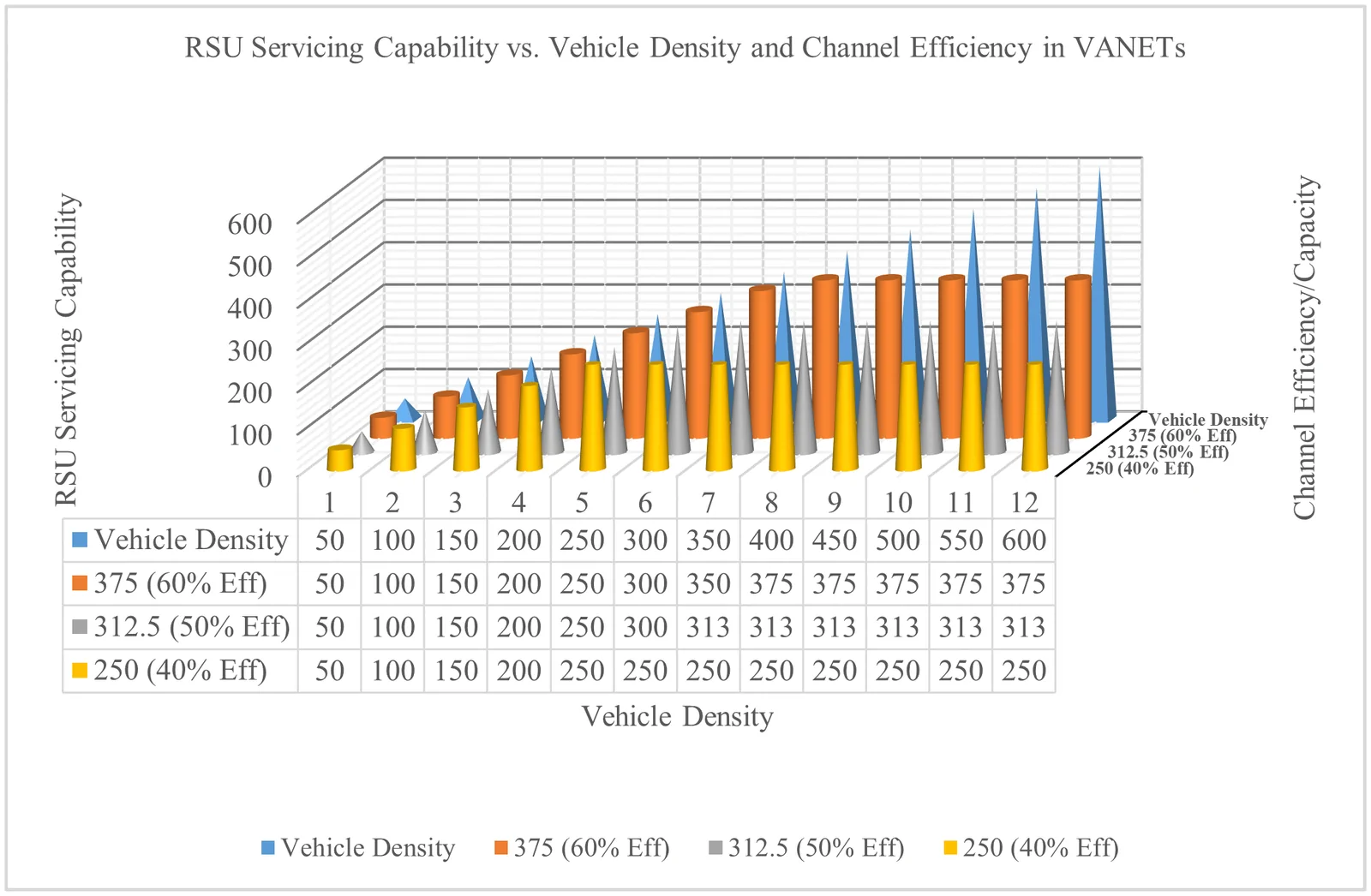
RSU Authentication and Service Rate vs. Vehicle Density under Varying Channel Efficiency Conditions.

Fig 8 illustrates the 3D grouped column chart for RSU (Roadside Unit) servicing capability as a function of vehicle density and channel efficiency in VANETs. The chart represents the number of vehicles that can be authenticated and served per second under three different channel efficiencies: 60% (cap = 375 vehicles/sec), 50% (cap = 312.5 vehicles/sec) and 40% (cap = 250 vehicles/sec). The X-axis shows increasing vehicle density (50–600 vehicles), the Y-axis corresponds to the RSU servicing capability (vehicles per second) and coloured bars indicate performance under each channel efficiency scenario.

**Major observations**:

- At low vehicle densities (<100), the RSU can serve all vehicles across all speeds.
- At medium densities (200–400), the channel becomes a limiting factor at higher speeds (due to reduced efficiency).
- At high densities (500–600 vehicles), channel capacity saturates, limiting the number of vehicles served, even though cryptographic capacity remains underutilized.
- The RSU CPU can handle over 31,000 auth/sec, so communication, not computation, is the primary bottleneck.

## 7. Analysis by NS-3 simulations

The proposed protocol was integrated into the NS-3 framework by modelling the authentication message exchange (D1, D2, D3, D4) as part of the application layer traffic. The computational delay for cryptographic operations presented in Table 6 was incorporated using a custom delay model to ensure realistic timing.

### 7.1. Packet Delivery Ratio (PDR)

**Fig 9:**
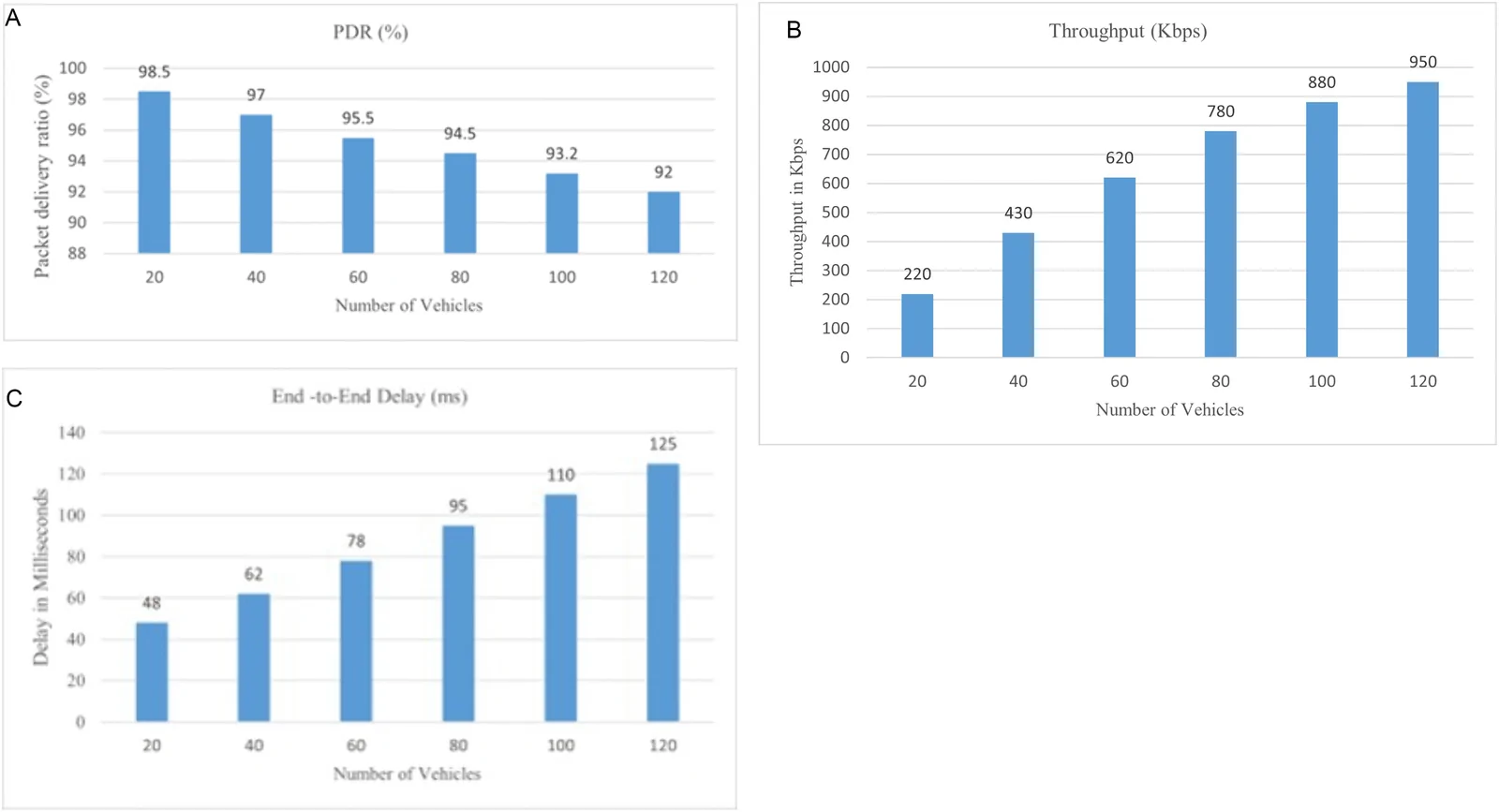
a) Packet delivery ratio, b) Throughput, c) End-to-end delay.

PDR is calculated as the ratio of the number of data packets successfully delivered to the destination to the number of packets generated by the source. Table 13 shows the simulation environment configuration parameters. The table lists all relevant settings including mobility model, network topology, vehicle density, transmission range and channel characteristics. It is observed that PDR slightly decreases from 98.5% to 92% when the vehicle density increases from 20 to 120. So, the PDR is always above 92 percent as shown in Fig 9a. This is true for 120 vehicles too. Too many vehicles cause network congestion. This may also cause packet collisions because of too many packets in transmission. Our handshake methodology is so light. It does not add too many control packets. So, the performance remains good.

**Table 13:** Simulation Parameters Notations.

Parameter	Value
Number of Vehicles	20 to 120 (in increments of 20)
Number of RSUs	4
Simulation Area	2000 m x 2000 m
Simulation Time	500 seconds
Mobility Model	Random Waypoint Mobility Model
Vehicle Speed	10 m/s to 30 m/s (randomly assigned)
MAC Protocol	IEEE 802.11p
Propagation Model	Two-Ray Ground
Traffic Type	Constant Bit Rate (CBR)
Data Packet Size	512 Bytes
Packet Rate	4 packets/second
Authentication Payload Size	As per proposed protocol (2976 bits)
Channel Data Rate	6 Mbps
NS-3 Version	3.35
QPSK	Quadrature Phase Shift Keying
OFDM	Orthogonal Frequency Division Multiplexing

### 7.2. Throughput

Throughput shows that more vehicles mean more throughput. In other words, the total amount of data successfully delivered to all the destination vehicles per unit of time. It is evident that throughput increases from 220 Kbps to 950 Kbps as more vehicles generate and forward data. Even though the individual packet delivery ratio drops slightly, the throughput does not change, as shown in Fig 9b.

### 7.3. End-to-end delay

This measure depicts the average travel time for a data packet. The packet goes from the source vehicle to the destination. The destination could be TA, user or RSU. This delay (as shown in Fig 9c) includes queuing delay, propagation delay, transmission delay and authentication processing delay. It is observed that the delay increases from 48 ms to 125 ms when the number of vehicles grows. It shows that they also have more queuing delays. Cryptographic operations add some standard fixed delay. This is a good trade-off for security.

The simulation results confirm our protocol is secure. It is also efficient and practical for real VANETs. The overhead is manageable. So, network performance stays strong even when the network grows.

## 8. Limitations and future work

In our scheme, some real messages with high latency may be rejected because their timestamps fall outside the ΔT window. Future work will fix this. We will use an adaptive tolerance range that can accept vehicles up to that range. It will change accordingly with network conditions. This will minimize false rejections but keep security. We will also study a blockchain-based TA for VANETs to make distributed TA services for decentralized key management. However, this paper does not study the distributed TA. The single point of failure remains unsolved. We leave this for the future scope.

## 9. Conclusion

In this article, we presented a novel anonymous authentication model using Chebyshev polynomials and a chaotic map, along with a normal matrix-based binary exponentiation algorithm. The proposed method handles the security challenges of VANETs, which come from their open and dynamic nature, by enabling efficient and secure Chebyshev polynomial calculations. Our scheme achieved 0.949468 ms execution time and 264 bytes of communication overhead. We used Scyther for formal verification. All 17 security claims are OK. This proves mutual authentication works, it proves key secrecy works, and it also proves replay attack resistance works. According to the theoretical study in our scheme, RSU can process 31,000 vehicles per second. Due to the practical density limit of the DSRC channel, each RSU can process 400 vehicles. We developed a fast authentication scheme specifically for VANETs, which reduces both computational and communication costs by using lightweight Chebyshev polynomials and hash functions during authentication and key agreement processes. This makes our approach more efficient than current framework solutions. As part of our future work, we intend to evaluate the energy consumption and resource utilization of the proposed authentication protocol through implementation on representative vehicular hardware and realistic VANET simulation environments. Moreover, we intend to evaluate the scalability of the proposed authentication protocol under high vehicle density and large-scale VANET environments through extensive simulations and real-world traffic scenarios.
